# Boride-derived oxygen-evolution catalysts

**DOI:** 10.1038/s41467-021-26307-7

**Published:** 2021-10-19

**Authors:** Ning Wang, Aoni Xu, Pengfei Ou, Sung-Fu Hung, Adnan Ozden, Ying-Rui Lu, Jehad Abed, Ziyun Wang, Yu Yan, Meng-Jia Sun, Yujian Xia, Mei Han, Jingrui Han, Kaili Yao, Feng-Yi Wu, Pei-Hsuan Chen, Alberto Vomiero, Ali Seifitokaldani, Xuhui Sun, David Sinton, Yongchang Liu, Edward H. Sargent, Hongyan Liang

**Affiliations:** 1 School of Materials Science and Engineering, Tianjin University, Tianjin, 300350 China; 2 Department of Electrical and Computer Engineering, University of Toronto, 35 St George Street, Toronto, Ontario M5S 1A4 Canada; 3 Department of Applied Chemistry, National Chiao Tung University, Hsinchu, 300 Taiwan ROC; 4 Department of Mechanical and Industrial Engineering, University of Toronto, 5 King’s College Road, Toronto, Ontario M5S 3G8 Canada; 5 National Synchrotron Radiation Research Center, Hsinchu, 30076 Taiwan ROC; 6 Institute of Functional Nano & Soft Materials (FUNSOM), Jiangsu Key Laboratory for Carbon-Based Materials and Devices, Soochow University, Suzhou, Jiangsu 215123 China; 7 Division of Materials Science, Department of Engineering Sciences and Mathematics, Luleå University of Technology, 97187 Luleå, Sweden; 8 Department of Molecular Sciences and Nanosystems, Ca’ Foscari University of Venice, Via Torino 155, 30172 Venezia Mestre, Italy; 9 Department of Chemical Engineering, McGill University, Montreal, Quebec H3A 0C5 Canada

**Keywords:** Electrocatalysis, Hydrogen energy, Electrocatalysis

## Abstract

Metal borides/borates have been considered promising as oxygen evolution reaction catalysts; however, to date, there is a dearth of evidence of long-term stability at practical current densities. Here we report a phase composition modulation approach to fabricate effective borides/borates-based catalysts. We find that metal borides in-situ formed metal borates are responsible for their high activity. This knowledge prompts us to synthesize NiFe-Boride, and to use it as a templating precursor to form an active NiFe-Borate catalyst. This boride-derived oxide catalyzes oxygen evolution with an overpotential of 167 mV at 10 mA/cm^2^ in 1 M KOH electrolyte and requires a record-low overpotential of 460 mV to maintain water splitting performance for over 400 h at current density of 1 A/cm^2^. We couple the catalyst with CO reduction in an alkaline membrane electrode assembly electrolyser, reporting stable C_2_H_4_ electrosynthesis at current density 200 mA/cm^2^ for over 80 h.

## Abstract (web summary)

Metal borides/borates are promising candidates to become high-performance alkaline oxygen evolution reaction catalysts. This study reports an in-situ phase composition modulation approach to fabricate boride/borate-based catalysts.

## Introduction

The design of active, stable, and cost-effective catalysts for the oxygen evolution reaction (OER) is critical to improving the full process efficiency in electrocatalytic systems, such as water splitting and CO_2_/CO reduction reaction (CO_2_/CORR) coupled with OER^1–4^. In these systems, approaches that use alkaline electrolytes have the benefit of increasing efficiency toward CO_2_RR and CORR by suppressing the competing hydrogen evolution reaction (HER).

While there have been improvements in the activity of metal phosphides, sulfides, selenides, and borides/borates^5–8^, achieving high electrocatalytic activity (>1 A/cm^2^) united with long-term stability (>100 h) remains an unmet need^9,10^. The lack of highly active and stable OER catalysts limits the performance and stability of electrosynthesis processes that rely on an OER anode (i.e., CO_2_RR/CORR electrolysers) to production rates <100 mA/cm^2^ and durations of <100 h. It remains an important task for the field to increase catalytic activity while achieving long-term durable and earth-abundant OER catalysts that operate at alkaline pH.

Metal borides/borates exhibit promising OER performance in high-pH regimes: incorporating highly electronegative boron diminishes the metal oxidation reaction energy barrier under a positive bias and assists with charge transfer^11,12^. To date, the durability of metal borides has been limited to <60 h at a modest current density of ~20 mA/cm^2,13–15^. Thus far, to improve the activity and stability, significant efforts have been invested in charge-transfer enhancement and electronic structure optimization; however, we found herein that metal borides are prone to convert into metal borate under OER conditions, and thus to behave as the *operando* catalyst^16–18^.

This knowledge prompted us to design metal borides as precursors to construct, in-situ, the metal borates that would endow the catalysts with activity and stability.

We focused on a NiFe-Boride compound and probed its phase transformation using *operando* X-ray spectroscopy during OER. We witnessed the in-situ transformation of NiFe-Boride to NiB_4_O_7_ and FeBO_3_ phases on the catalyst surface during electrochemical activation. These results accord with DFT calculations which reveal that NiB_4_O_7_ species facilitate the step *O - > *OOH and enhance electrocatalytic activity toward OER.

Experimentally, we achieve an overpotential of 167 mV at 10 mA/cm^2^ and stable water splitting performance of 400 h at 1 A/cm^2^. Encouraged by its high activity and stability, we implement NiFe-Boride as the OER catalyst in a CORR membrane-electrode assembly (MEA) electrolyser that uses CO and H_2_O to produce C_2_H_4_^19^. The CORR electrolyser based on the NiFe-Boride OER catalyst exhibited stable performance at 200 mA/cm^2^ for over 80 h—maintaining a C_2_H_4_ energy efficiency of 19%—whereas this value is 17% for the CORR electrolyser that relies on a noble IrO_2_ OER catalyst.

## Results

### Insight from computational studies into stable and active phases

**Fig. 1:**
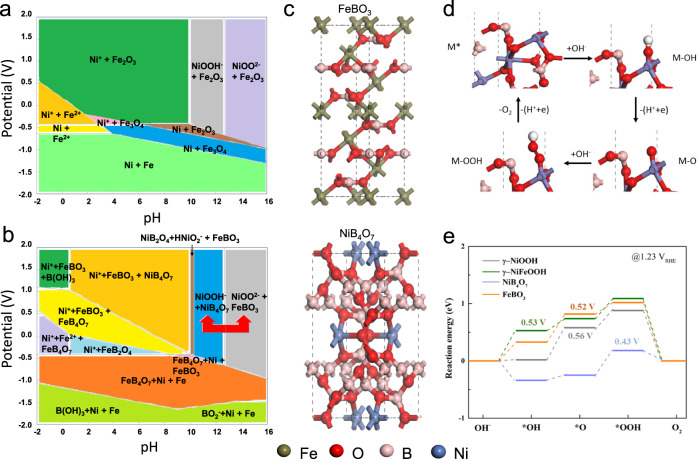
Results of DFT calculations for NiFe-Boride catalysts. Pourbaix diagram of (**a**) NiFe and (**b**) NiFe-Boride; (**c**) The cell configurations of NiB_4_O_7_ and FeBO_3_; (**d**) The schematic illustration of the 4-step OER pathway; (**e**) Predicted OER reaction energy diagram for NiB_4_O_7_, FeBO_3_, γ-NiOOH, and γ-NiFeOOH in the alkaline electrolyte at 1.23 V vs. RHE.

Noting that metal borides undergo phase transformation at highly oxidative potentials, we sought to calculate Pourbaix diagrams to evaluate the phase composition of NiFe-Boride ternary system at high potentials in alkaline electrolytes. Compared to the Pourbaix diagram of NiFe (Fig. 1a), we observe two new stable phases, FeBO_3_ and NiB_4_O_7_, under a working potential (*U* = 1.4—2 V vs. RHE) at electrolyte pH (pH = 10—14) of alkaline OER in the Pourbaix diagram of NiFe-Boride (Fig. 1b), indicating that boron ions promote the new phases formation. The atomic configurations of these two new phases are shown in the Fig. 1c.

We carried out density functional theory (DFT) calculations to further explore the effects of these in-situ formed phases on the OER activity of NiFe catalyst. We first calculated the reaction energy of elementary steps and the overpotential for the OER based on the 4e-mechanism proposed by Nørskov et al. for water oxidation^20–22^ with the reaction pathway:

1 $$\ast +{{{{{{\rm{OH}}}}}}}^{-}\to \ast {{{{{\rm{OH}}}}}}+{e}^{-}$$

2 $$\ast {{{{{\rm{OH}}}}}}+{{{{{{\rm{OH}}}}}}}^{-}\to \ast {{{{{\rm{O}}}}}}+{{{{{{\rm{H}}}}}}}_{2}{{{{{\rm{O}}}}}}+{e}^{-}$$

3 $$\ast {{{{{\rm{O}}}}}}+{{{{{{\rm{OH}}}}}}}^{-}\to \ast {{{{{\rm{OOH}}}}}}+{{{{{{\rm{e}}}}}}}^{-}$$

4 $$\ast {{{{{\rm{OOH}}}}}}+{{{{{{\rm{OH}}}}}}}^{-}\to {{{{{{\rm{O}}}}}}}_{2}+{{{{{{\rm{H}}}}}}}_{2}{{{{{\rm{O}}}}}}+{{{{{{\rm{e}}}}}}}^{-}$$

We cleaved the (100) facet of these two new phases to study the adsorption energy of various intermediates for OER, and thus to determine the theoretical overpotentials as an indicator for catalytic activity (Fig. 1d and Supplementary Fig. 1). The largest reaction energy (Step 2, deprotonation of *OH) was identified as the potential-determining step (PDS)^23^, as shown in Fig. 1e, and the reaction energy was similar for both FeBO_3_ (0.53 V) and NiOOH (0.56 V) at 1.23 V. In contrast, NiB_4_O_7_ shows a thermodynamic overpotential of 0.43 V, lowest among NiOOH, NiFeOOH and FeBO_3_, which suggests that NiB_4_O_7_ phases are the active sites in NiFe-Boride that enable superior catalytic OER performance. We observed that the NiB_4_O_7_ shifts the PDS to the *OOH formation step at 1.23 V. We also obtained the similar calculation results at 1.40 V (Fig. 1e and Supplementary Fig. 2).

To account for other possible reaction mechanisms, we further studied the intramolecular oxygen coupling mechanism^24^: we found that Ni and Fe borate have increased activity compared to Ni and Fe (oxy)hydroxide (Supplementary Fig. 3). We also considered more sophisticated activity descriptors for multiple-electron processes, exploring the use of electrochemical-step symmetry index (ESSI) and the PDS at zero overpotential (G_max_)^25–27^: these results indicated higher OER activities of these NiFe-borate phases (Supplementary Table 1). Taken together, these results indicate that boron addition enables formation of new active phases, especially the Ni phase, in the pristine framework, thereby enhancing electrocatalytic activity toward OER.

### Catalyst synthesis and characterization

**Fig. 2:**
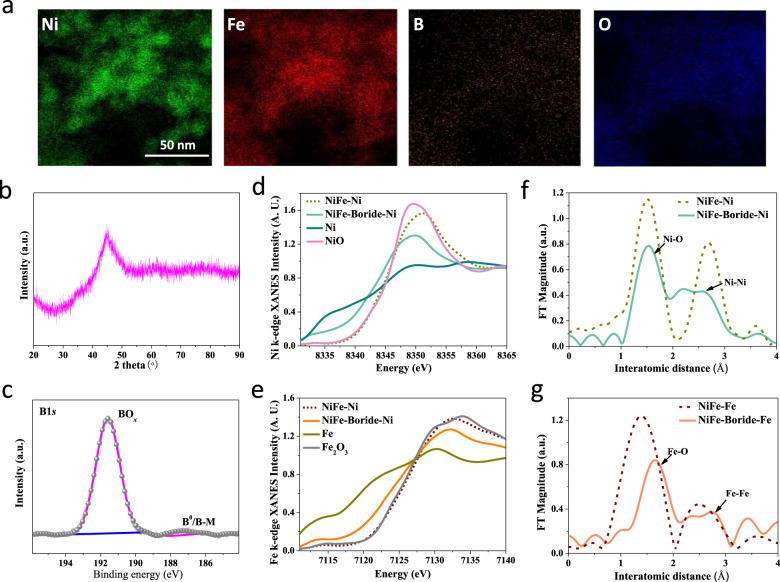
Characterization of the morphology and composition of NiFe-Boride catalyst. (**a**) Elemental mapping analysis, showing the uniform, uncorrelated spatial distribution of Ni, Fe, B and O. (**b**) XRD patterns. (**c**) B 1 *s* XPS spectrum of fresh NiFe-Boride sample. The Ni and Fe *K*-edge XANES (**d**) and (**e**) and EXAFS (**f**) and (**g**) data for fresh NiFe-Boride and control samples.

To test these hypotheses experimentally, we synthesized NiFe-Boride catalysts by a chemical reduction method (see Methods for details). Transmission electron microscopy (TEM), scanning electron microscopy (SEM), and X-ray diffraction (XRD) were conducted to investigate the structure, composition, and morphology of the NiFe-Boride catalyst. TEM and SEM images (Supplementary Figs. 4 and 5) revealed that catalysts the consisted of agglomerated nanoparticles. Elemental mapping analysis indicated that Ni, Fe, B, and O elements distribute homogeneously, without obvious separation or aggregation (Fig. 2a and Supplementary Fig. 6). A broad XRD diffraction peak between 40° and 50° indicates low crystallinity in the catalyst (Fig. 2b).

We further carried out X-ray photoelectron spectroscopy (XPS) to study the chemical state of the NiFe-Boride catalyst. XPS survey spectra show the presence of Ni, Fe, B, and O, which agrees well with the elemental mapping results (Supplementary Fig. 7). The B 1 *s* spectrum obtained from the pristine sample was comprised of two peaks located at 192 eV and 187 eV, which are assigned to B-O and B^0^/B-M species (Fig. 2c), respectively, indicating boride. The Ni and Fe *K*-edge X-ray absorption near-edge structure (XANES, Fig. 2d, e) revealed that NiFe-Boride possesses an electronic structure that differs from that of the control samples, indicating that the incorporation of boride leads to phase and structural changes. To probe the origins of these changes, we obtained a Fourier transform (FT) of the extended X-ray absorption fine structure (EXAFS, Fig. 2f, g) spectrum. NiFe-Boride showed two FT features: (1) the peak at ~1.5 Å represents the single-scattering path of M-O and (2) the peak at ~2.5 Å results from the single-scattering path of the closest neighboring transition metals around the metal cations (M-M) (the locations of the peaks match the locations of those in the NiFe control sample)^28^. The altered electronic structure suggests that the boride ions were incorporated into the NiFe framework via the formation of BO_*x*_ and NiFeB_*x*_ species. Further investigations of the M-B peak remain a topic of interest.

Using inductively coupled plasma optical emission spectrometry (ICP-OES) analysis, we determined the molar ratio of Ni:Fe to be 1:1. Further analysis of the as-deposited system with the aid of energy-dispersive X-ray spectroscopy (EDS) indicated a 1:1 atomic ratio of Ni:Fe (Supplementary Fig. 8).

### In-situ and ex-situ characterization

**Fig. 3:**
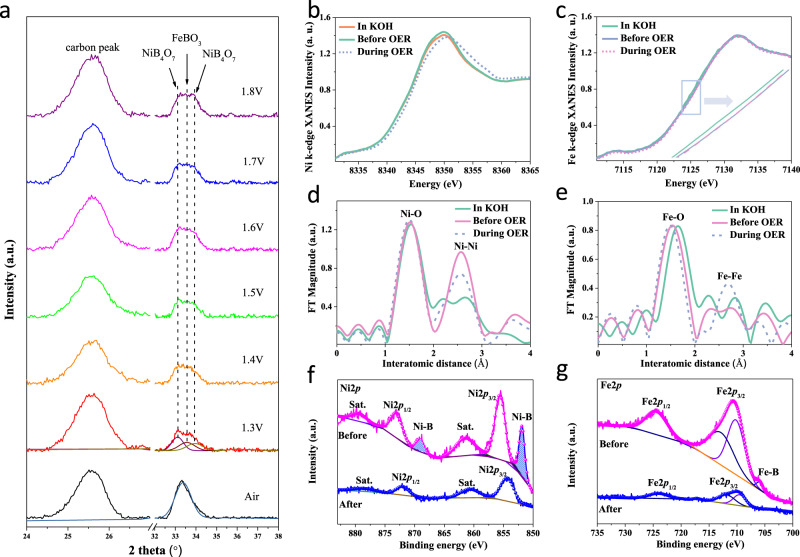
Ex/in-situ characterization of as-prepared NiFe-Boride catalyst. **a** In-situ SRXRD patterns of the catalyst during the OER test in 1 M KOH electrolyte; The in-situ Ni and Fe *K*-edge XANES (**b**) and (**c**) and EXAFS (**d**) and (**e**) data of NiFe-Boride during the LSV process in 1 M KOH aqueous electrolyte. The in-situ applied voltages before and during the OER are 1.2 and 1.4 V vs. RHE, respectively. **f** Ni 2*p* and (**g**) Fe 2*p* XPS spectra for fresh and post-OER samples.

Using in-situ synchrotron radiation X-ray diffraction (SRXRD), we tracked changes in the crystal structure of catalysts during the OER process. We found that, prior to OER, the phase structure of the catalyst was NiFeBO_4_ (see Supplementary Fig. 9 for the model structure). We found that FeBO_3_ and NiB_4_O_7_ were formed under an applied potential (Fig. 3a, the standard patterns are provided in the Supplementary Fig. 10), consistent with in-situ formation of new phases in the NiFe-Boride catalyst during OER. We then carried out in-situ XAS to monitor the local electronic structure changes of the metal sites during the OER. The Ni and Fe *K*-edge XANES (Fig. 3b, c) spectra exhibited a positive energy shift, i.e., the valence states of Ni and Fe increased with the applied potential^29^. We analyzed the absorption edge energies in order to characterize the Ni/Fe valence states. Before OER, the valence states of Ni and Fe in NiFe-Boride were 1.91 and 2.73, respectively. During OER, the valence states of Ni and Fe increased to 1.98 and 2.95, respectively (Supplementary Figs. 11–13). The *ex-situ L*-edge XAS results were in good agreement with this change: the reacted catalyst – compared to the pristine one – had a higher Ni^3+^ ratio and a similar Fe^2+/3+^ ratio. The similarity in Fe^2+/3+^ ratios indicates that Fe ions return to their initial states upon release of the applied potential. These results accord with the higher Ni^3+^ ratio in the reacted catalyst observed via O *K*-edge XAS (Supplementary Fig. 14).

We further analyzed the EXAFS spectra (Fig. 3d, e) to clarify the local structural variations associated with the Ni and Fe sites^30,31^. Under open circuit potential (OCP) in KOH electrolyte, the FT peaks demonstrated features similar to those obtained from the as-prepared catalysts. Once a positive bias was applied, the progressive FT peaks show that the original phase accomplishes some structural transformations. We conducted EXAFS fitting (Supplementary Tables 2–3 and Supplementary Figs. 15, 16) to determine the bond lengths of M-O and M-M (M is Ni or Fe). The interatomic distances of Ni-O and Ni-Ni(Fe) in as-prepared NiFe-Boride are 2.04 Å and 3.09 Å, respectively, while those measured during OER are 2.02 Å and 2.98 Å. The interatomic distances of Fe-O and Fe-Fe(Ni) in as-prepared NiFe-Boride are 2.23 Å and 3.22 Å, while those measured during OER are 1.92 and 3.05 Å. As oxidation and phase transformation progress, the M-O and M-M bonds become shorter than those at OCP. These results are in line with the generation of transition metal active sites with high valence^29^. The electronic structure of NiFe-Boride is different than that of Ni-Boride and Fe-Boride, indicating the presence of electronic communication between the Ni and Fe in M-M bonds (Supplementary Fig. 17).

We carried out XPS to study the local evolution of chemical state and electronic structure before vs. after OER, with all peaks calibrated using C 1 *s* (Supplementary Fig. 18). High-resolution XPS in Ni 2*p* region showed two main peaks of Ni^2+^ 2*p*_3/2_ and Ni^2+^ 2*p*_1/2_ located at 855.4 and 873.3 eV (Fig. 3f), accompanied by satellite peaks centered at 861.2 and 879.4 eV^32^. In the Fe 2*p* region, two main peaks located at 710.7 and 724.6 eV were assigned to Fe 2*p*_3/2_ and Fe 2*p*_1/2_ (Fig. 3g). We noted minor peaks centered at ~851.9 and 706.1 eV in the Ni 2*p* and Fe 2*p* spectra of fresh catalyst, suggesting the existence of Ni-B and Fe-B bonds in NiFe-Boride (Fig. 3f, g)^33^, consistent with EXAFS. The B 1 *s* spectrum consists of two peaks corresponding to B-O and B^0^/B-M species. Upon completion of electrochemical activation during OER, negative chemical shifts in the binding energy of Ni 2*p* and Fe 2*p* peaks were observed compared to pristine NiFe-Boride. These negative shifts indicated the modulation of the surface electronic structure through the oxidation process, in accordance with the positive shift in the peak position of B-O bonds (Supplementary Fig. 19). Harsh oxidative conditions during OER converted the M-B species into oxides and hydroxides, evidenced by the disappearance of Ni-B, Fe-B, and B^0^/B-M signals.

In sum, NiFe-Boride acts as a precursor that phase transforms at highly oxidative potentials, leading to the formation of stable FeBO_3_ and NiB_4_O_7_ phases during the OER process.

### Electrocatalytic performance

**Fig. 4:**
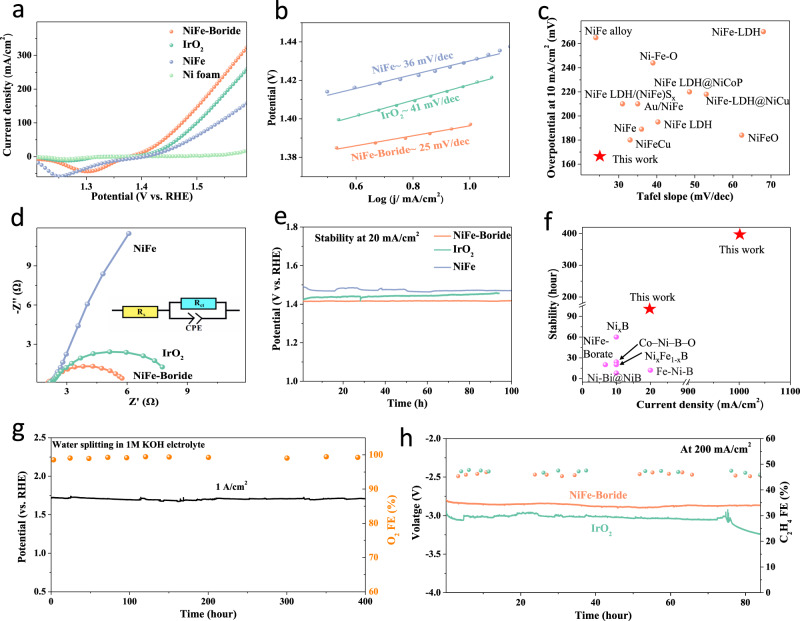
Performance of NiFe-Boride catalyst and controls in a three-electrode configuration in 1M KOH aqueous electrolyte. **a** OER LSV polarization curves for catalysts loaded on Ni foam without *iR* correction. **b** The corresponding Tafel plot of catalysts. **c** Comparison of Tafel slope and overpotential required to achieve 10 mA/cm^2^, with references all measured in alkaline medium^47–57^. **d** EIS data for NiFe-Boride and controls in three-electrode configuration. The data were collected at 1.45 V vs. RHE. The inset provides the equivalent circuit: R_*s*_ series resistance, R_*ct*_ charge-transfer resistance, and CPE constant-phase element related to the double-layer capacitance. **e** Chronopotentiometric curves obtained from the NiFe-Boride catalyst on Ni foam electrode at a constant current density of 20 mA/cm^2^. **f** Comparison of stability at different current density, with references all measured in alkaline medium^13–15,33,58^. **g** Operating voltage and O_2_ FE at constant 1 A/cm^2^ current density in a three-electrode configuration in 1 M KOH aqueous electrolyte. **h** The operating voltage and ethylene FE were monitored at constant 200 mA/cm^2^ in a membrane-electrode assembly device. NiFe-Boride and IrO_2_ supported on titanium felt were used as the anodes. The high surface area Cu catalyst on hydrophobic carbon paper acted as cathode. Humidified CO was flowed through the gas channels in the cathode, and 2 M aqueous KOH solution was flowed through channels in the anode.

The OER performance of NiFe-Boride and controls on Ni foam was examined in 1 M KOH electrolyte using a three-electrode cell at room temperature (see Methods). As shown in linear sweep voltammetry (LSV, Fig. 4a) curves, the overpotential of NiFe-Boride catalyst at 10 mA/cm^2,34^ and without *iR* compensation is 167 mV. This is lower than the 200 mV and 189 mV obtained for sol-gel NiFe and IrO_2_, respectively (Supplementary Table 4). Rotating ring disk measurements show a similar trend: NiFe-Boride enables higher-rate water oxidation performance than in control catalysts (Supplementary Fig. 20). To seek insight into the effect of in-situ active sites on performance, we compared the catalytic activity between annealed and pristine catalysts. The annealed catalysts have lower performance, consistent with an activity-promoter role for the in-situ formed active sites (Supplementary Figs. 21-22). A Ni:Fe ratio of 1:1 (Supplementary Fig. 23 and Table 5) leads to the lowest overpotential. We determined the Tafel slope (Fig. 4b), finding 25 mV/dec for the NiFe-Boride catalyst, lower than for IrO_2_ (41 mV/dec) and NiFe (66 mV/dec). The lower Tafel slope value indicating rate-determining step is shifted from the *O step to the *OOH formation step^35,36^. Compared to benchmark OER catalysts from literature, NiFe-Boride shows higher catalytic performance in terms of overpotential at 10 mA/cm^2^ and Tafel slope in the alkaline solution (Fig. 4c). From Nyquist plots based on electrochemical impedance spectroscopy (EIS, Fig. 4d) at applied potential 1.45 V vs. RHE reveal that NiFe-Boride exhibits a lower charge-transfer resistance (R_*ct*_) than does either NiFe or IrO_2_.

We then used electrochemically active surface area (ECSA) obtained via the double-layer capacitance (*C*_*dl*_, Supplementary Fig. 24) technique to estimate a normalized current density^37^. This for NiFe-Boride is 7.5 mA/cm^2^, >2x greater than that in IrO_2_, and 4x greater than in NiFe catalysts, at 1.45 V vs. RHE (Supplementary Fig. 25). *IR*-corrected LSV excludes the effect of ionic conductivity on performance: the NiFe-Boride catalyst showed an *iR*-corrected overpotential of 160 mV at 10 mA/cm^2^ on Ni foam (Supplementary Fig. 26), which is 35 and 23 mV lower than for NiFe and IrO_2_.

We measured the stability of the best-performing NiFe-Boride catalyst at 20 mA/cm^2^ and found that the increase in potential was less than 10 mV after 100 h of continuous water splitting (Fig. 4e), more stable than the control and reference samples (Fig. 4f). We obtained stability at 1 A/cm^2^, finding that catalysts enable stable operation of 400 h with an overpotential of 460 mV without degradation (Fig. 4g). B 1 s XPS results indicate that the B concentration gradually decreases over the first 100 h but then remains constant over the ensuing 300 h (Supplementary Fig. 27). The Faradaic efficiency (FE) toward O_2_ remains 98 ± 2% over the first 400 h, excluding a major current contribution associated with materials corrosion.

To investigate further the potential relevance of these catalysts to future systems, we implemented NiFe-Boride as the OER catalyst in a CORR MEA electrolyser that produces C_2_H_4_ from CO and H_2_O (Supplementary Fig. 28). The CORR electrolyser exhibited stable performance at 200 mA/cm^2^ for over 80 h, maintaining a cell potential of −2.85 V, a C_2_H_4_ FE of 45%, and a C_2_H_4_ energy efficiency of 19%. Under similar operating conditions, the cell potential for the IrO_2_ OER catalyst-based electrolyser was −3.06 V, which is ~200 mV higher than in the NiFe-Boride OER catalyst-based system (Fig. 4h and Supplementary Fig. 29), corresponding to a C_2_H_4_ energy efficiency of 17%. Using XPS and EDS line scanning, we found that Ni, Fe, and B elemental signals remained following the stability study (Supplementary Figs. 30 and 31)., i.e., the borate remained in the NiFe.

## Discussion

Starting from DFT calculations, we saw that the phase transformation of NiFe-Boride to NiFe-Borate can be activated under OER potentials in OER. The in-situ formed new phase (NiB_4_O_7_) can function as active site to facilitate *O - > *OOH, enhancing the electrocatalytic activity of NiFe-Boride in OER. We evaluated the electrochemical performance of the catalyst and found that NiFe-Boride possesses a Tafel slope of 25 mV/dec and an overpotential of 167 mV at current density 10 mA/cm^2^ in 1 M KOH solution, outperforming benchmark NiFe oxide. The in-situ formed NiFe-Borate phases are confirmed via *operando* spectroscopies. The catalyst demonstrates stable performance over 400 h at 1 A/cm^2^ in water splitting. We further showcased 80 h of stable C_2_H_4_ electrosynthesis using a NiFe-Boride OER catalyst in a CORR MEA electrolyser, with a ~200 mV lower cell potential compared to MEA electrolysers based IrO_2_ OER catalysts.

## Methods

### I.1. Chemicals

Iron (III) chloride (FeCl_3_), nickel (II) chloride hexahygrate (NiCl_2_·6H_2_O), Sodium borohydride (NaBH_4_), ethanol (≥99.5%) and acetone (≥99 %) were purchased from Sigma-Aldrich. Iridium (IV) oxide dehydrate (99.99%) was purchased from Alfa Aesar^®^. All chemicals were used without further purification.

### I.2. Synthesis of NiFe-Boride and control catalysts

Th NiFe-Boride catalysts were synthesized using a modified chemical reduction method. NiCl_2_·6H_2_O (332.8 mg) and anhydrous FeCl_3_ (227.1 mg) were first dissolved in deionized water (DI, 2 mL) in a vial. A solution of NaBH_4_ (2 mL, 5 M) in water was prepared in a separate vial. All solutions mentioned above were cooled in an ice bath for 10 min to prevent uncontrolled hydrolysis and consideration which may lead to the formation of the precipitate. The Ni and Fe precursors were then added dropwise to NaBH_4_ to form a black solution. Finally, the prepared solution was further washed and centrifuged with water and ethanol three times before vacuum drying. The samples of different Ni:Fe ratios were synthesized by changing the Ni and Fe precursors concentration.

NiFe catalysts were synthesized using a modified aqueous sol-gel technique. NiCl_2_·6H_2_O (2.1 mmol) and anhydrous FeCl_3_ (0.35 mmol) were first dissolved in ethanol (2 mL) in a vial. A solution of deionized water (DI) (0.18 mL) in ethanol (2 mL) was prepared in a separate vial. All solutions mentioned above were cooled in an ice bath for 2 h to prevent uncontrolled hydrolysis and consideration, which may lead to the formation of precipitate rather than gel formation. The Ni and Fe precursors were then mixed with an ethanol-water mixture to form a clear solution. Propylene oxide (≈ 1 mL) was then slowly added to form a gel. Finally, the prepared NiFe wet-gel was aged for 24 h to promote network formation and immersed in acetone for 5 days before the gel was vacuum dried.

### I.3. Characterization

The morphologies of the catalysts were characterized by SEM (Hitachi S-4800) imaging, and energy-dispersive X-ray spectroscopy mapping operated at 1.5 kV was used to characterize the surfaces of all samples. TEM images and corresponding elemental mapping analysis were conducted using a JEM-2100F. The electronic structure measurements were carried out using XPS (ESCALAB 250Xi). To determine the best Ni/Fe ratio in the NiFe-Boride system, we first dissolved 50 mg of the catalyst into 5 ml aqua regia through microwave digestion. Then, we took out 1 ml of sample and diluted it to 10 mL to test ICP-OES (5110).

In-situ XAS was carried out in the same condition as electrocatalytic characterization case using a modified single cell with an opening sealed by Kapton tape. The XAS signal was collected by Vortex detector in 9BM beamline of Advanced Photon Source (APS) in Argonne National Laboratory. X-ray absorption near-edge spectra (XANES) of Ni and Fe *K*-edge were collected by silicon drift detector at ambient air in 9BM beamline of Advanced Photon Source (APS) located in the Argonne National Laboratory.

In-situ SRXRD measurements on NiFe-Boride in a typical three-electrode setup as the same condition in electrochemical characterization case were conducted at beamline 01C2 at National Synchrotron Radiation Research Center using a specially designed acrylic container with a 1 mL of gap, sealed by Kepton tape. 16 keV of the incident X-ray (λ = 0.7749 Å) was allowed to perpendicularly transmit through the sample and electrolyte, so that the signal of synchrotron X-ray diffraction of transmission mode would be collected in a large Debye-Scherrer camera and processed using the GSAS-II software 45 to obtain plots of intensity versus 2θ.

### I.4. Catalysts loaded on the electrodes

To load the catalyst on the Ni foam (thickness: 1.6 mm, Sigma), 20 mg of catalyst was dispersed in a mixture consisting of 2 mL water and 2 mL ethanol, followed by the addition of 100 μL Nafion solution. The suspension was sonicated for 30 min to prepare a homogeneous ink. Ni foam with a fixed area of 0.5 × 0.5 cm^2^ coated with water-resistant silicone glue was drop-casted with 20 μL of the catalyst ink. Ag/AgCl (with saturated KCl as the filling solution) and platinum foil were used as the reference and counter electrodes, respectively.

### I.5. Electrochemical characterization

Electrochemical measurements were performed using a three-electrode system connected to an electrochemical workstation (Autolab PGSTAT302N), equipped with built-in EIS analyzer. The working electrode was Ni foam (thickness: 1.6 mm, Sigma), which was prepared by dispersing 20 mg of catalyst in a mixture containing 1 mL water and 1 mL ethanol, followed by the addition of 100 μL Nafion solution. The suspension was sonicated for 30 min to prepare a homogeneous ink. Cyclic voltammetry (CV) measurements at 50 mV/s were performed prior to recording LSV at 1 mV/s for each sample. EIS measurements were conducted in a static solution at 1.45 V (vs. RHE). The amplitude of the sinusoidal wave was 10 mV, and the frequency scan range was between 100 kHz and 0.01 Hz. All experiments were performed at ambient temperature (23 ± 2 °C), and electrode potential was converted to the RHE scale using Eq. (5)

5 $$E\left({vs}.{RHE}\right)=E({vs}.{Ag}/{AgCl})+{E}_{{Ag}/{AgCl}}({vs}.{NHE})+0.059\times {pH}$$

### MEA test

The MEA cell is a complete CO electrolyser (Dioxide Materials) consisted of a titanium anode flow field, 904 L Stainless Steel cathode flow field, and associated nuts, bolts and insulating kit. The geometric area of each flow field is 5 cm^2^, of which 45% is the channel while the rest 55% is the land area. The catalyst electrode was attached on the cathode by a copper tape, which was protected by insulating Kapton tapes to avoid electrical contact with membranes or electrolytes. A Sustainion^®^ AEM membrane (Fumasep) was activated in 1 M aqueous KOH solution for 24 h, washed with water prior to use. The anode consisted of NiFe-Boride supported on titanium felt was prepared by a dip-coating and thermal decomposition method. The cathode is the high surface area Cu catalyst on hydrophobic carbon paper. Aqueous 2 M KOH electrolyte was used as the anolyte and was circulated using a peristaltic pump. The flow rate of the anolyte was kept at 20 mL/min. The flow rate of the CO gas flowing into the cathodic gas chamber was kept at 80 sccm by a mass flow controller. The CORR was initiated by applying a constant current density of 200 mA/cm^2^, and the current density was kept constant over the course of electrolysis.

### IR correction

All the polarization curves on different supports were corrected for ohmic losses (including the wiring, substrate, catalyst, and solution resistances). The *iR*-corrected data were given by the following equation:

6 $${E}_{{Co}{rrected}}=E-{iR}$$

where *R* is the series resistance of measurement, which can be obtained from an EIS Nyquist plot as the first intercept of the main arc with the axis.

### ECSA

The ECSA of each catalyst was determined by measuring the electrochemical double-layer capacitances (*C*_*dl*_) from the scan rate CV-dependence plot. The CV cycle potential window was between 0.7 and 0.8 V vs. RHE, and the scan rates were 20, 40, 60, 80, 100, and 120 mV/s. The *C*_*dl*_ was estimated $\triangle j=\frac{1}{2}(j_{{charge}}-{J}_{{off\; charge}})$ at the average potential in the selected range against the scan rates. The slope of the linear fit was calculated as the *C*_*dl*_. A specific capacitance of 40 μF/cm^2^ was used here. The ECSA of the catalyst was calculated by using the following equation,

7 $${ECSA}=\frac{{C}_{{dl}}}{40\mu F/{{cm}}^{2}}{{cm}}_{{ECSA}}^{2}$$

### DFT calculations

We performed the DFT calculations using the Vienna Ab Initio Simulation Package (VASP) code^38,39^. The exchange-correlation energy was modelled using Perdew-Burke-Ernzerhof (PBE) functional^40^ within the generalized gradient approximation (GGA). The projector augmented-wave (PAW) pseudo-potentials^41^ were used to describe the ionic cores. The cut-off energy of 450 eV was adopted after a series of tests. A Methfessel–Paxton smearing of 0.05 eV to the orbital occupation was applied for the geometry optimization and total energy computations. In all calculations, atoms at all positions were assumed to possess Hellmann-Feynman forces lower than 0.02 eV/Å, and the electronic iteration convergence criterion was 10^-5^ eV using the normal algorithm. Pourbaix diagrams were calculated using Atomic Simulation Environment (ASE)^42^ with input formation energy values from Materials project^43^. Surface models for NiB_4_O_7_ and FeBO_3_ were simulated by five-layer (2 × 2) NiB_4_O_7_ (100) and FeBO_3_ (100) supercells (Supplementary Fig. 32), accompanied with a sufficient vacuum gap of 15 Å. Structural optimizations were performed on all modified slab models. We considered 4-steps intermediates adsorption to simulate the OER process (details in below & Supplementary Fig. 33). During adsorption calculations, the top two layers were fully relaxed, while the other layers were fixed at the tested lattice position.

The reaction energies (ΔG) were obtained by

8 $$\varDelta {{{{{\rm{G}}}}}}=\varDelta {{{{{{\rm{E}}}}}}}^{{{{{{\rm{DFT}}}}}}}+\varDelta {{{{{\rm{ZPE}}}}}}-{{{{{\rm{T}}}}}}\varDelta {{{{{\rm{S}}}}}}$$

where ΔE^DFT^ is the reaction energy calculated from DFT; ΔZPE is the zero-point energy, which was neglected here; ΔS is the change in entropy, and for gaseous molecules ΔS values are obtained from the standard database in the NIST web-book^44^.

Taking the reaction (1) as an example, the reaction energy is equals to

9 $$\varDelta {{{{{{\rm{G}}}}}}}^{1}={{{{{\rm{G}}}}}}(\ast {{{{{\rm{OH}}}}}})\mbox{-}{{{{{\rm{G}}}}}}(\ast )\mbox{-}G({{{{{{\rm{OH}}}}}}}^{-})$$

where G(*OH) is the total energy of *OH adsorption configuration; G(*) is the energy of catalyst surface; G(OH^-^) is the energy of hydroxyl ion, to calculate this value, we assume the equilibrium

10 $${H}_{2}{{{{{\rm{O}}}}}}\leftrightarrow {H}^{+}+{{OH}}^{-}$$

which relates the chemical potentials as

11 $${\mu }_{{H}^{+}}+{\mu }_{{{OH}}^{-}}={\mu }_{{H}_{2}{{{{{\rm{O}}}}}}}$$

Then,

12 $${\mu }_{{H}^{+}}+{\mu }_{{e}^{-}}+{\mu }_{{{OH}}^{-}}-{\mu }_{{e}^{-}}={\mu }_{{H}_{2}{{{{{\rm{O}}}}}}}$$

Thus,

13 $${\mu }_{{{OH}}^{-}}-{\mu }_{{e}^{-}}={\mu }_{{H}_{2}{{{{{\rm{O}}}}}}}-({\mu }_{{H}^{+}}+{\mu }_{{e}^{-}})$$

here, ${\mu }_{{H}^{+}}+{\mu }_{{e}^{-}}$ can be calculated using computational hydrogen electrode (CHE) model developed by Nørskov and co-workers^45,46^. Then, the theoretical overpotential $\eta$ for OER can be calculated by

14 $$\eta ={{{{{\rm{max }}}}}}\left(\Delta {G}^{i}\right)-1.23$$

$\Delta {G}^{i}$ stands for the reaction energy for reaction (8) – (11), *i* = 1, 2, 3, 4.

Electrochemical-step symmetry index (ESSI) and maximum energy G_max_ can act as a sophisticated activity descriptors for multiple-electron processes. We calculated ESSI using the following formula,

15 $${ESSI}=\frac{1}{n}\mathop{\sum }\limits_{k=1}^{n}\left(\frac{\triangle {G}_{k}^{+}}{e}\right)$$

where $\triangle {G}_{k}^{+}$ means all positive values of reaction energies, while negative values are not accounted.

The G_max_ is the free-energy spanning from intermediate with the smallest free energy to the intermediate with the highest energy during OER.

## Supplementary information

SI-basic OER

## Data Availability

The data supporting this study are available within the paper and the Supplementary Information. The source data of DFT calculations are provided in this paper. All other relevant source data are available from the corresponding authors upon reasonable request. Source data are provided with this paper.

## Source data

source data
