# Supplementary material for: Boride-derived oxygen-evolution catalysts
Source: Nat Commun. 2021 Oct 19;12:6089. doi: 10.1038/s41467-021-26307-7 (PMC8526748; doi:10.1038/s41467-021-26307-7)
Supplement: Supplementary file 1 — SI-basic OER [file 41467_2021_26307_MOESM1_ESM.pdf]

Supplementary Information for

Boride-Derived Oxygen-Evolution Catalysts

Ning Wang[1,2†], Aoni Xu[2†], Pengfei Ou[2†], Sung-Fu Hung[3†], Adnan Ozden[4], Ying-Rui Lu[5], Jehad Abed[2], Ziyun Wang[2], Yu Yan[2], Meng-Jia Sun[2], Yujian Xia[6], Mei Han[1], Jingrui Han[1], Kaili Yao[1], Feng-Yi Wu[3], Pei Hsuan Chen[3], Alberto Vomiero[7,8], Ali Seifitokaldani[9], Xuhui Sun[6], David Sinton[3], Yongchang Liu[1*], Edward H. Sargent[2*] and Hongyan Liang[1*]

[†]These authors contributed equally to this work. *Correspondence and requests for materials should be addressed to Edward H. Sargent (ted.sargent@utoronto.ca) (E.H.S), Yongchang Liu (ycliu@tju.edu.cn) (Y.C.L) and Hongyan Liang (hongyan.liang@tju.edu.cn) (H.Y.L)

This file includes:

Supplementary Figures 1 to 33

Supplementary Tables 1 and 5

References

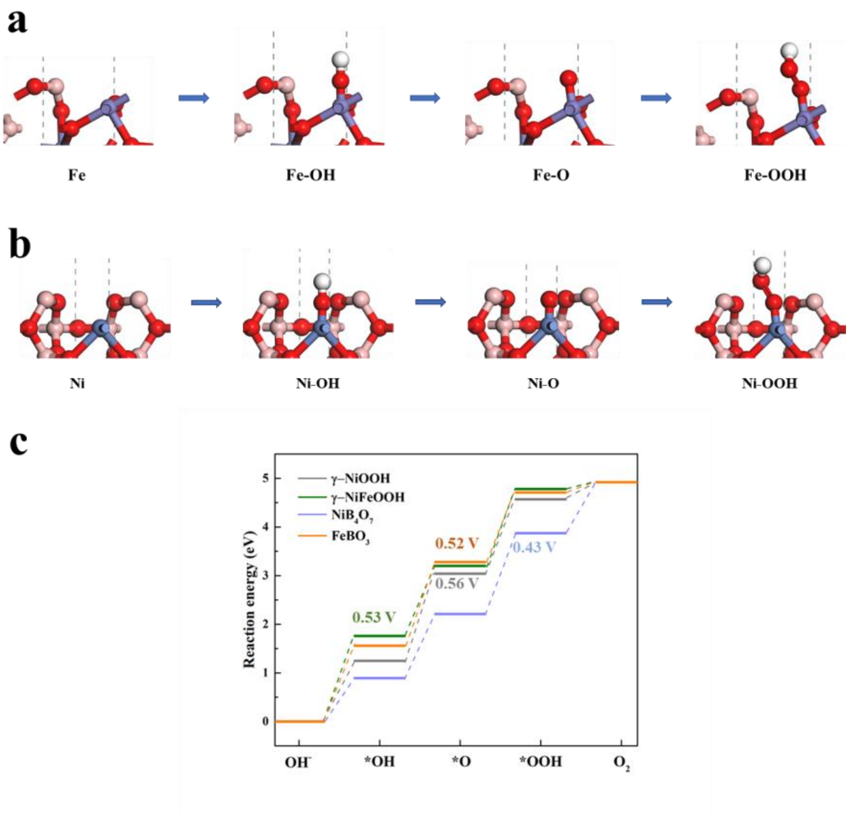

Supplementary Figure 1. Atomic reaction pathway of OER on (a) $FeBO_3$ and (b) NiB_4O_7 via water hydrogen atom abstraction mechanism. (c) Predicted OER reaction energy diagram for NiB_4O_7, $FeBO_3$ and NiOOH, NiFeOOH in alkaline electrolyte at 0 V.

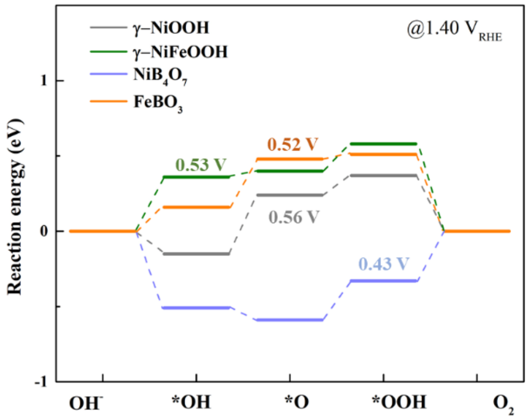

Supplementary Figure 2. Predicted OER reaction energy diagram for NiB_4O_7, $FeBO_3$ and NiOOH, NiFeOOH in alkaline electrolyte at 1.40 V vs RHE.

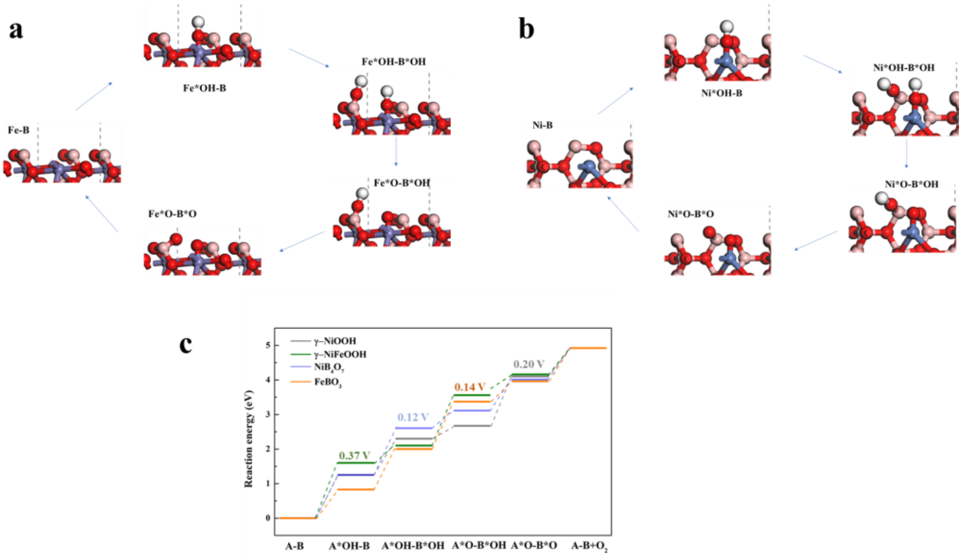

Supplementary Figure 3. Atomic reaction pathway of OER on (a) $FeBO_3$ and (b) NiB_4O_7 via intramolecular oxygen coupling mechanism. (c) Predicted OER reaction energy diagram for NiB_4O_7, $FeBO_3$ and NiOOH, NiFeOOH in alkaline electrolyte at 0 V.

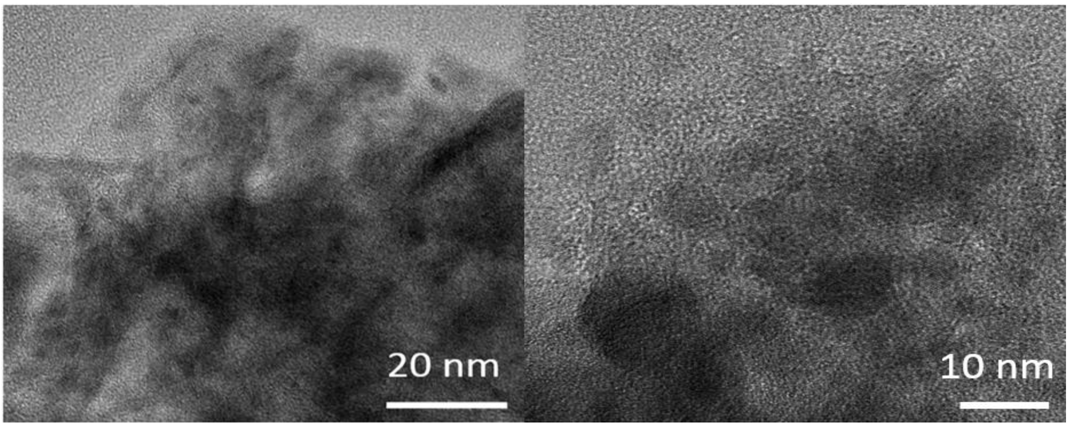

Supplementary Figure 4. Surface TEM images of NiFe-Boride catalyst.

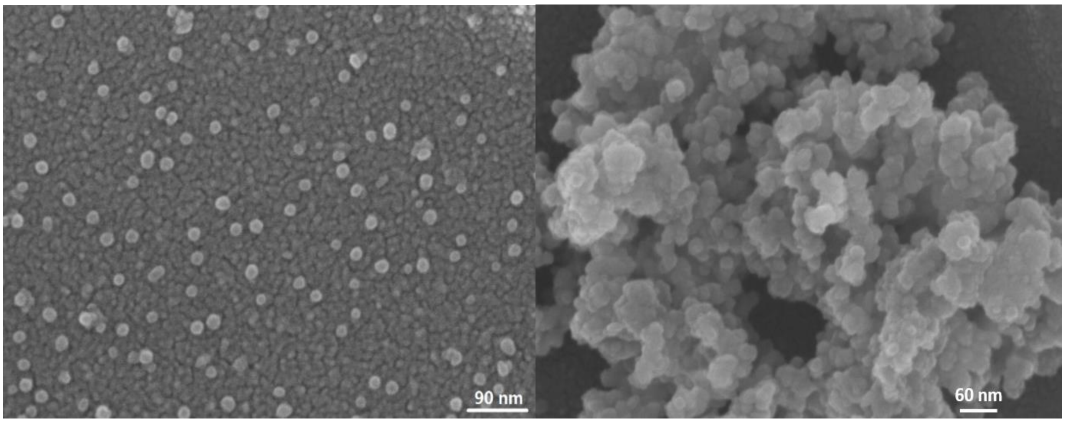

Supplementary Figure 5. Surface SEM images of NiFe-Boride catalyst.

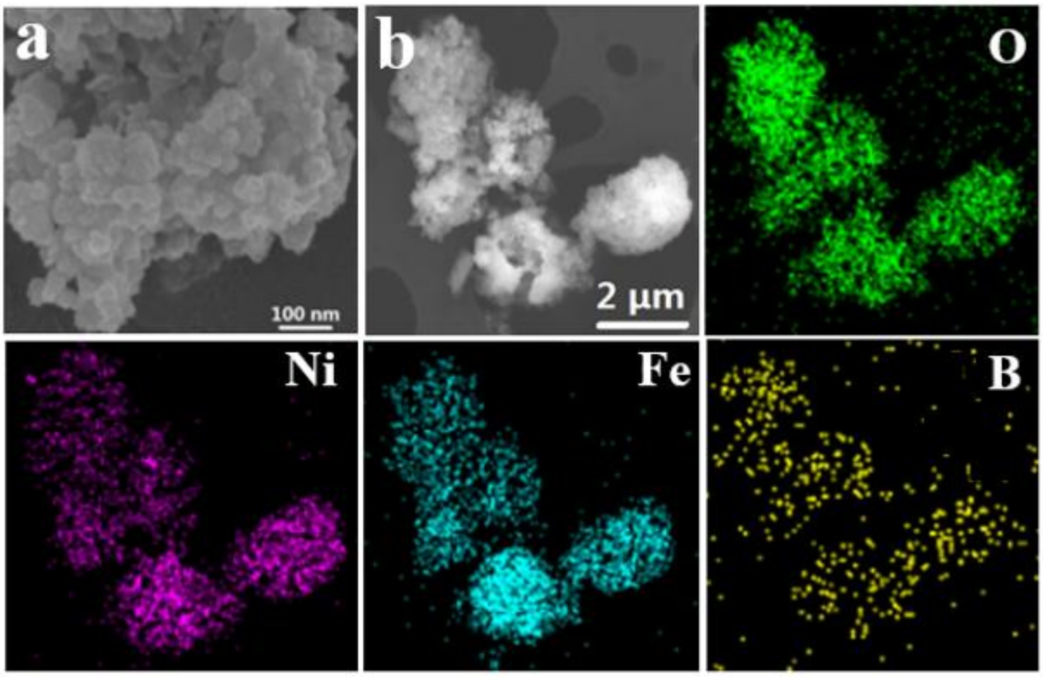

Supplementary Figure 6. Morphological and compositional characterization of NiFe-Boride catalyst. Representative of (a) SEM and (b) Elemental mapping of NiFe-Boride catalyst.

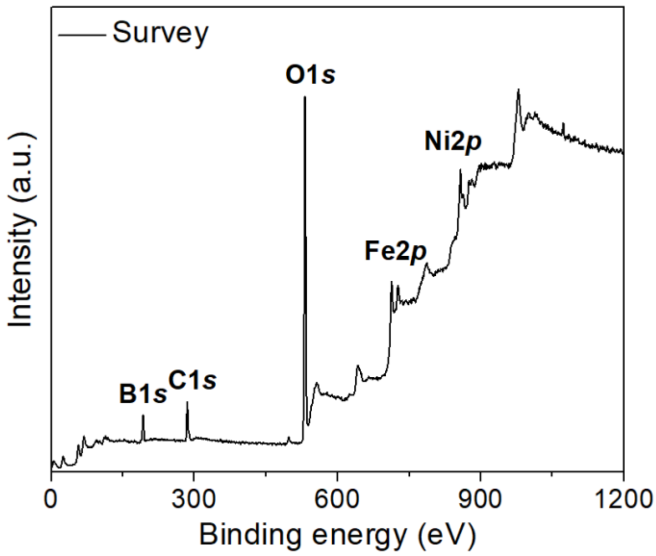

Supplementary Figure 7. The survey XPS spectrum of NiFe-Boride catalyst.

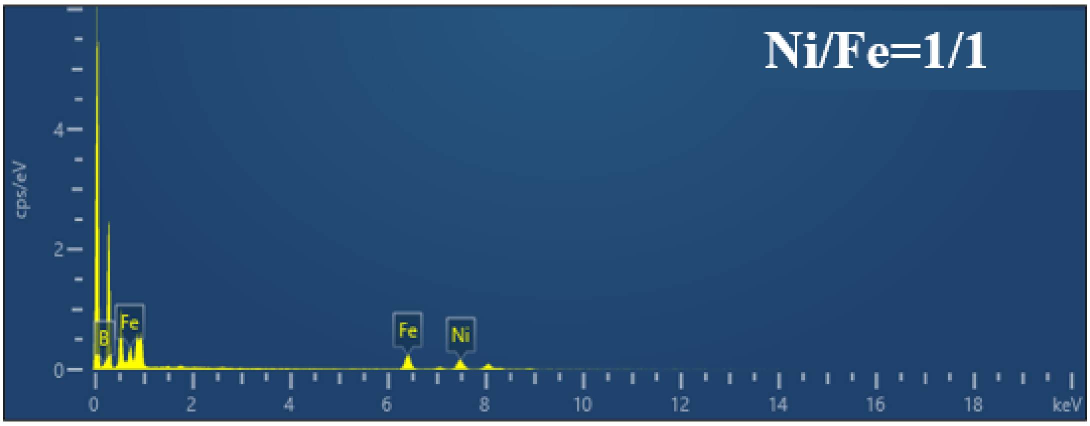

Supplementary Figure 8. EDX spectra of NiFe-Boride catalyst.

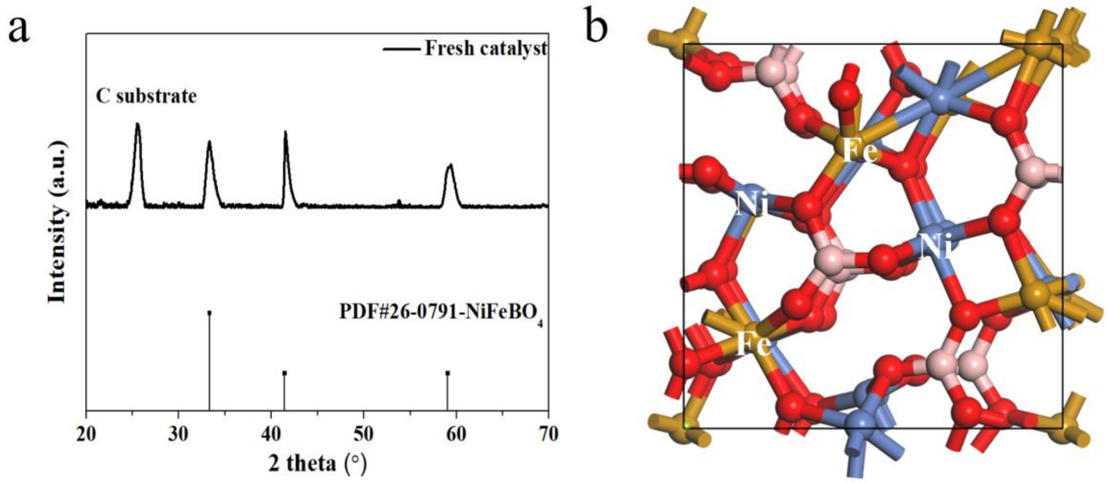

Supplementary Figure 9. (a) XRD pattern of fresh catalyst and (b) the model structure of $NiFeBO_4$.

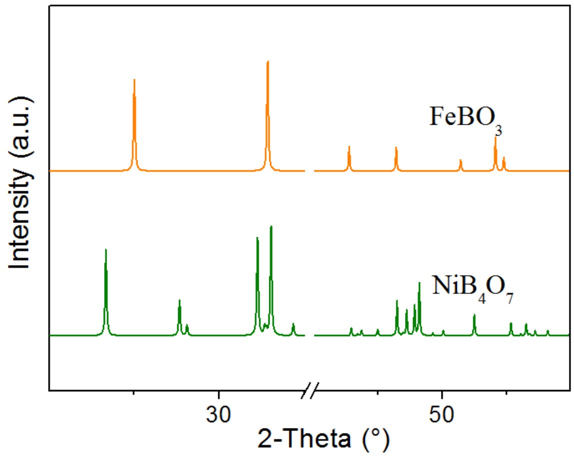

Supplementary Figure 10. The standard XRD patterns of NiB_4O_7 and $FeBO_3$.

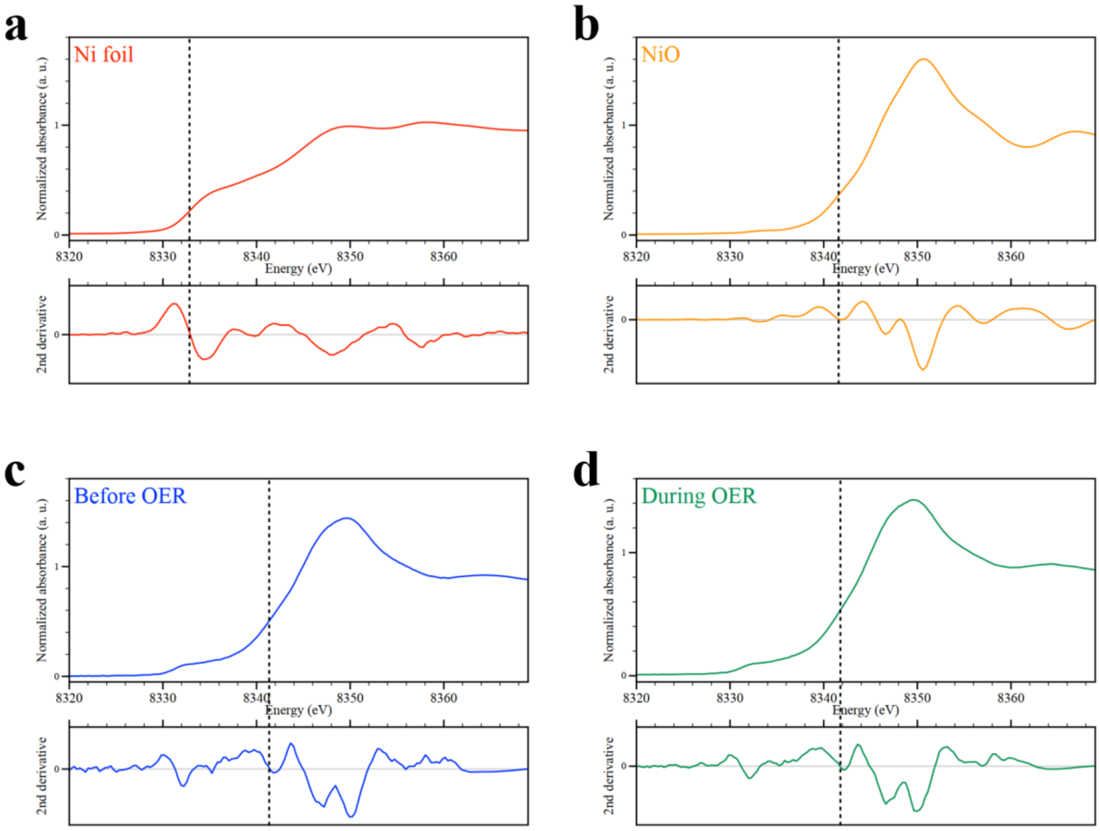

Supplementary Figure 11. *In-situ* sample characterization under electrocatalytic reaction conditions. Ni *K*-edge XANES spectra and second-order derivatives of the XANES spectra for the (a) Ni foil; (b) NiO and NiFe-Boride catalysts (c) before and (d) during OER process.

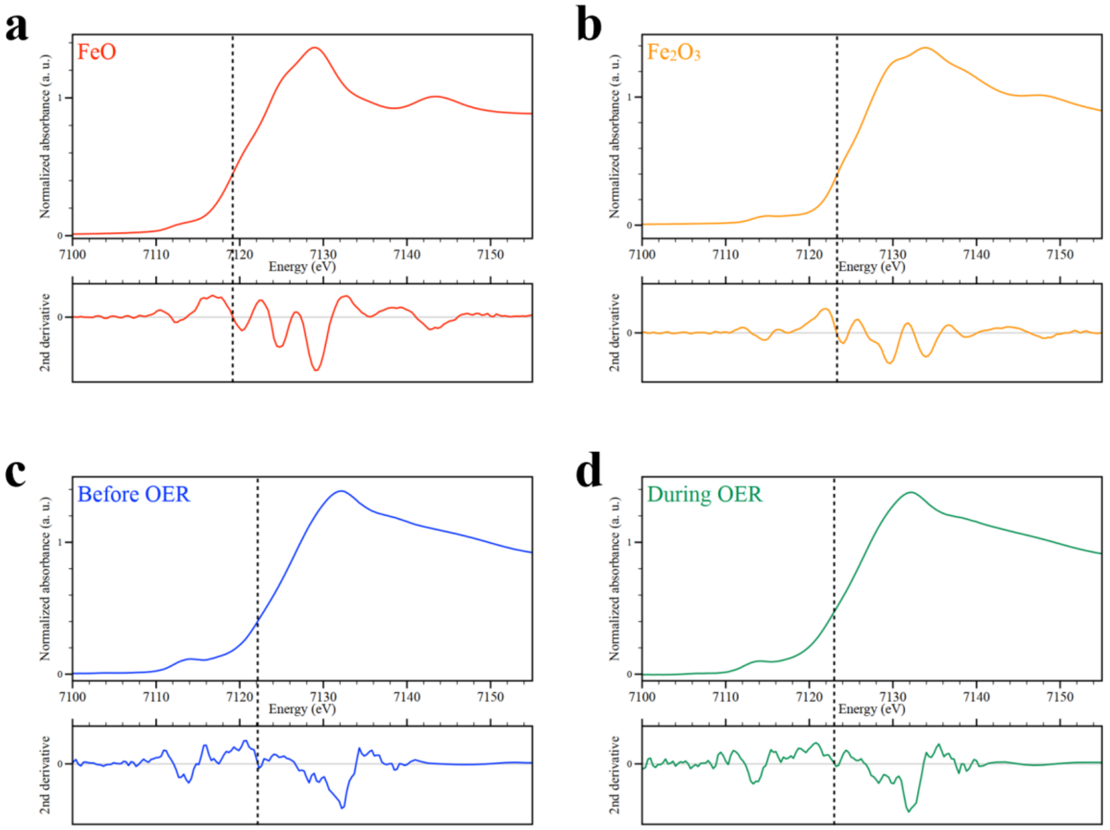

Supplementary Figure 12. *In situ* sample characterization under electrocatalytic reaction conditions. Fe *K*-edge XANES spectra and second-order derivatives of the XANES spectra for the (a)FeO; (b) Fe_2O_3 and NiFe-Boride samples (c) before and (d) during OER process.

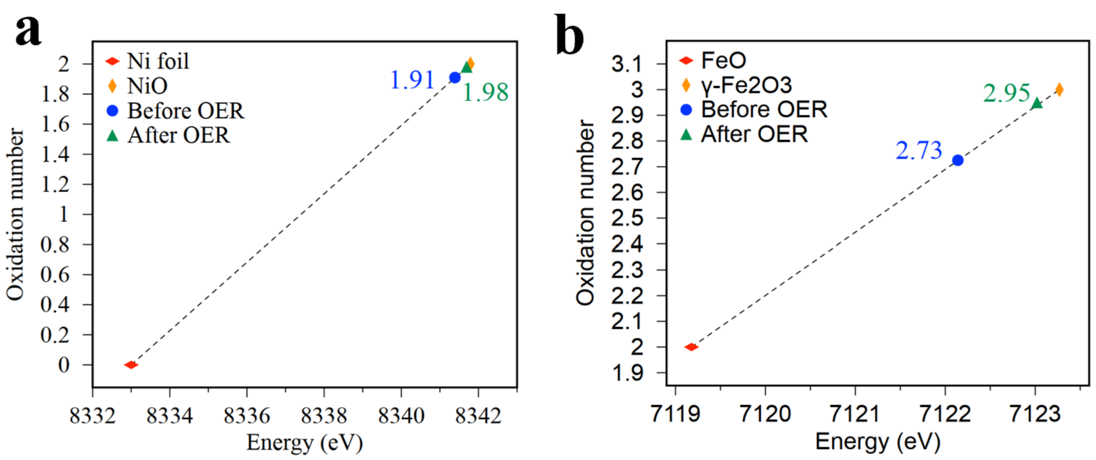

Supplementary Figure 13. *In situ* valence state change under electrocatalytic reaction conditions for NiFe-Boride and control catalysts. (a) Ni and (b) Fe valence.

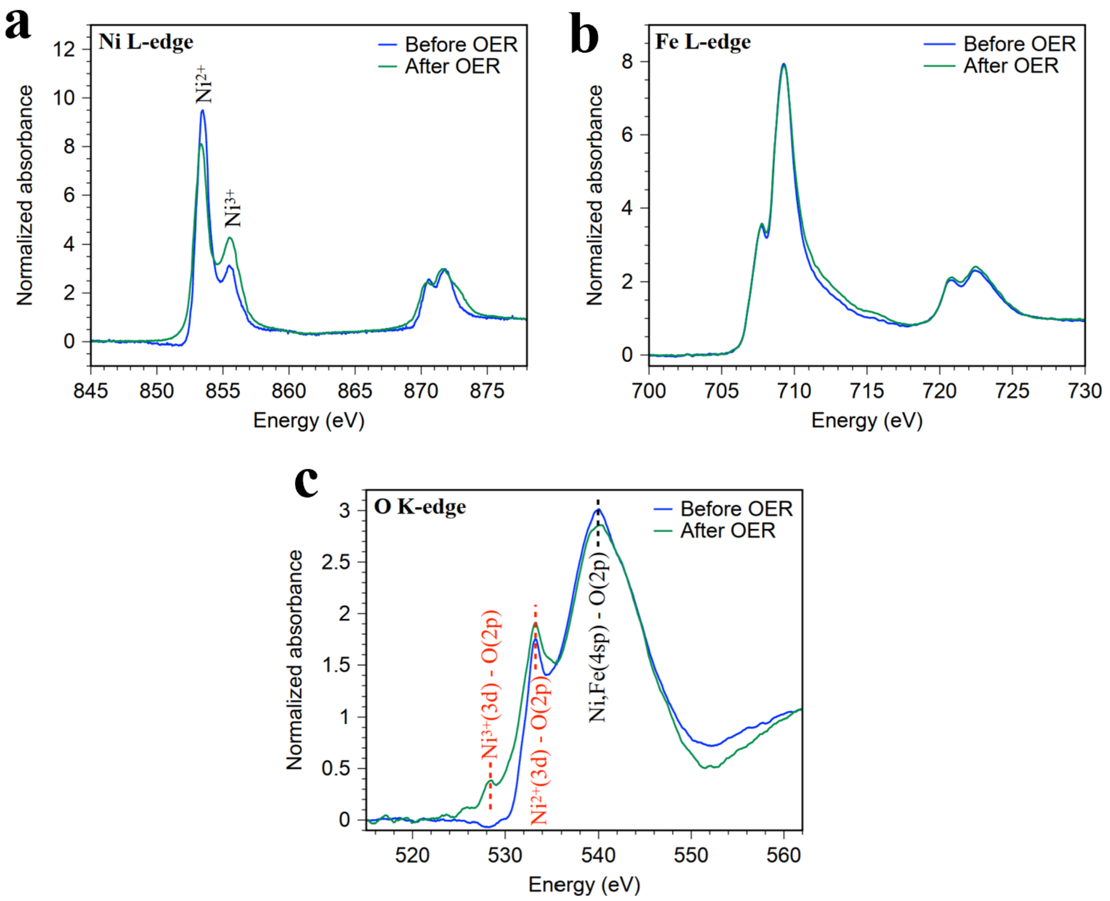

Supplementary Figure 14. *Ex-situ* (a) Ni *L*-edge, (b) Fe *L*-edge and (c) O *K*-edge spectra before and after the OER process for NiFe-Boride catalyst.

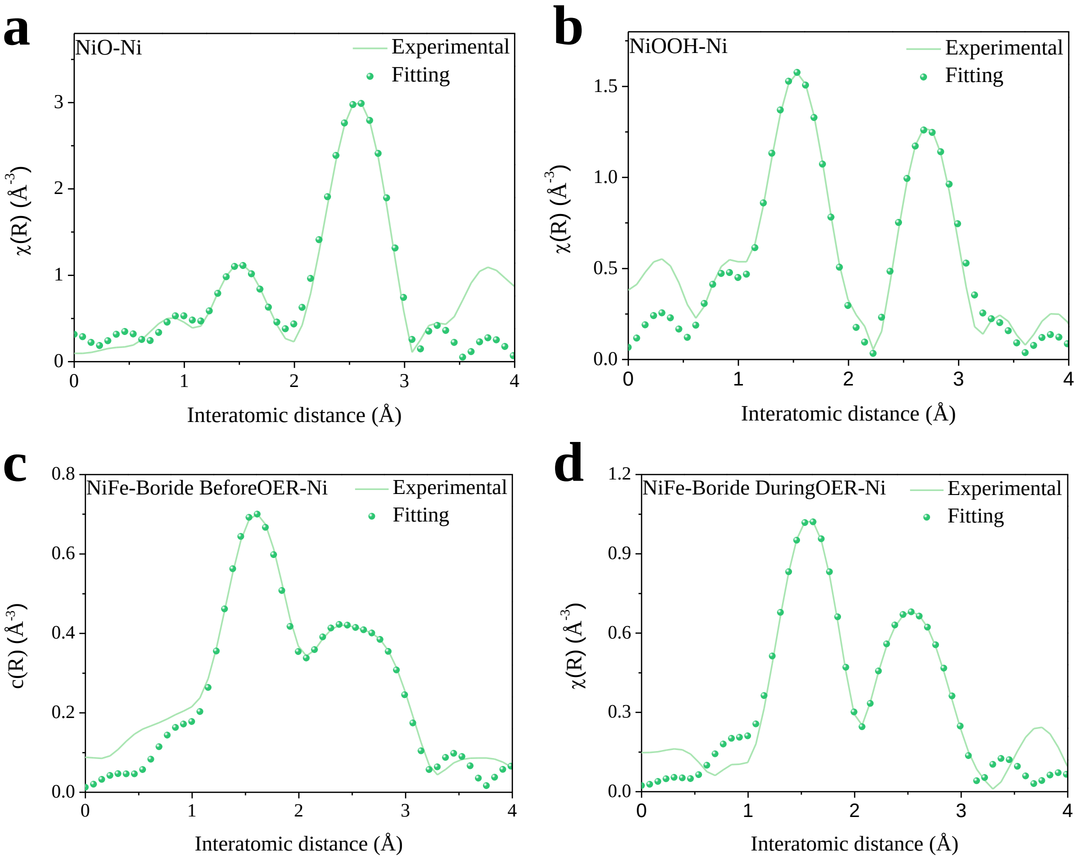

Supplementary Figure 15. EXAFS spectra and fitting of Ni *K*-edge from (a) NiO, (b) NiOOH, (c) NiFe-Boride before the OER process (d) NiFe-Boride after the OER process.

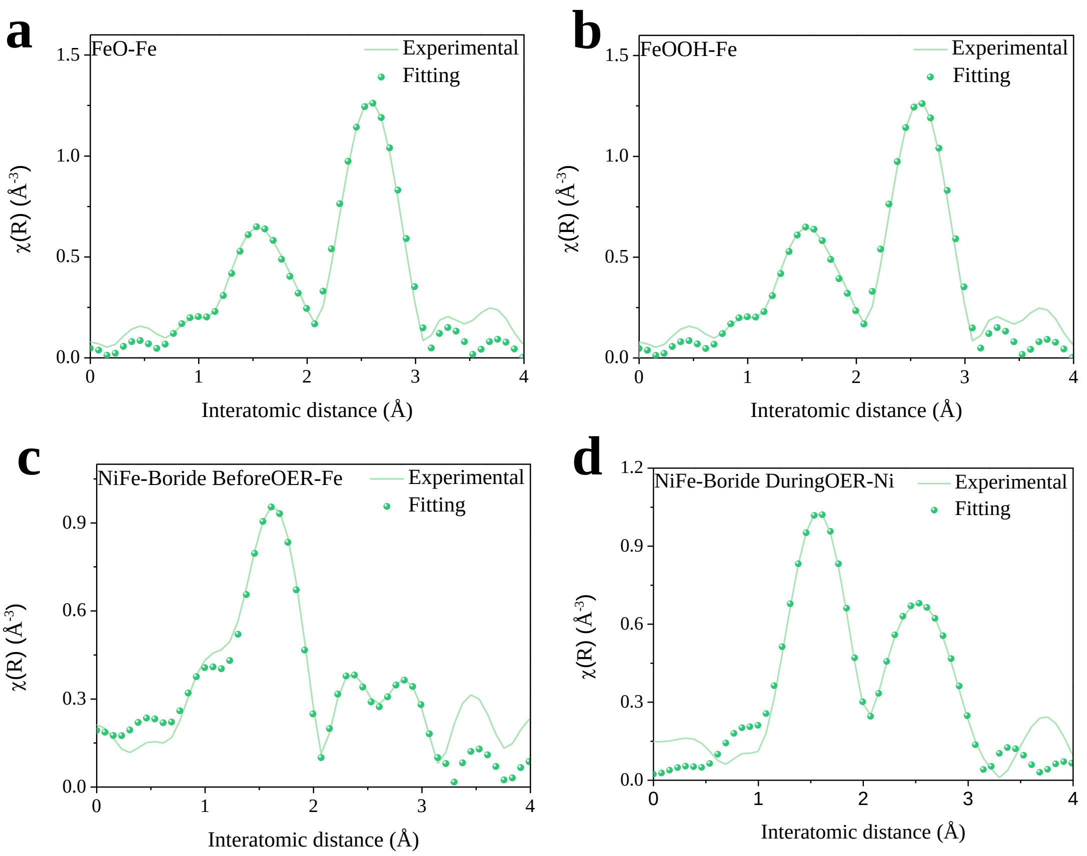

Supplementary Figure 16. EXAFS spectra and fitting of Fe *K*-edge from (a) FeO, (b) FeOOH, (c) NiFe-Boride before the OER process (d) NiFe-Boride after the OER process.

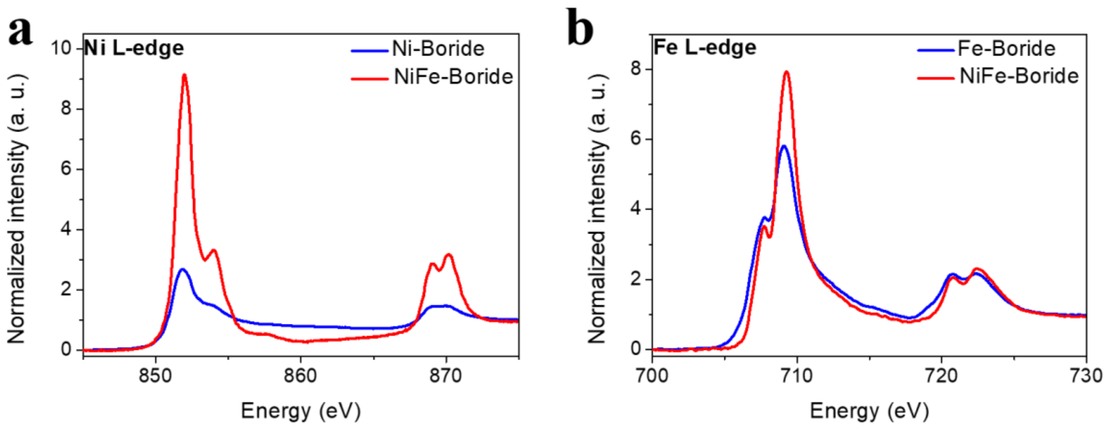

Supplementary Figure 17. *Ex-situ* (a) Ni and (b) Fe *L*-edge spectra for NiFe-Boride, Ni-Boride and Fe-Boride catalysts.

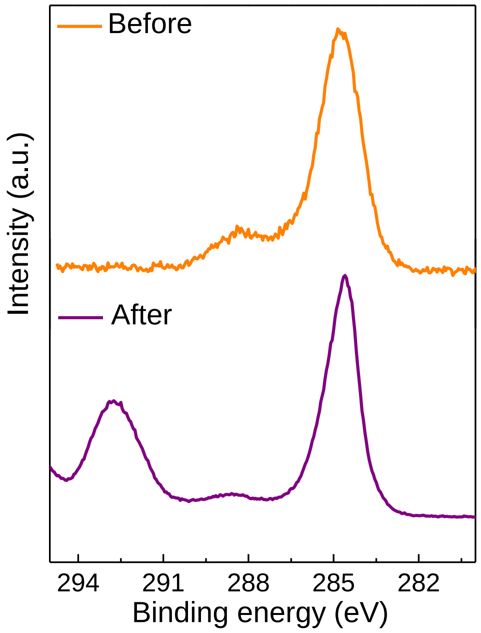

Supplementary Figure 18. The high-resolution C1*s* XPS spectra of NiFe-Boride catalysts before and after the activation.

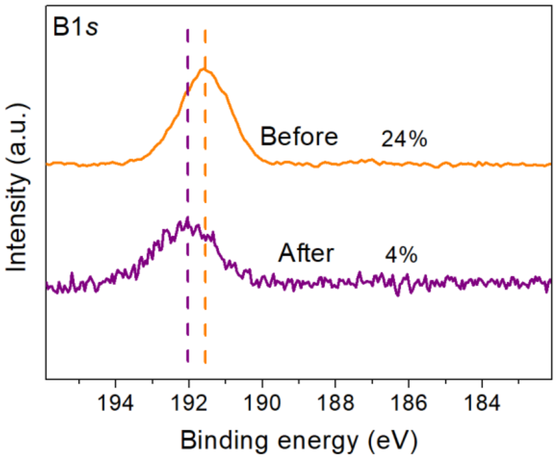

Supplementary Figure 19. The high-resolution B1*s* XPS spectra of NiFe-Boride catalysts before and after the activation.

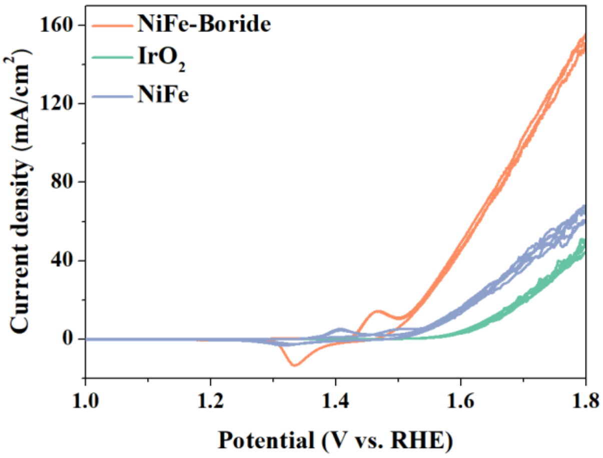

Supplementary Figure 20. OER LSV polarization curves for NiFe-Boride and control catalysts on rotating ring disks in a three-electrode configuration in 1 M KOH aqueous electrolyte.

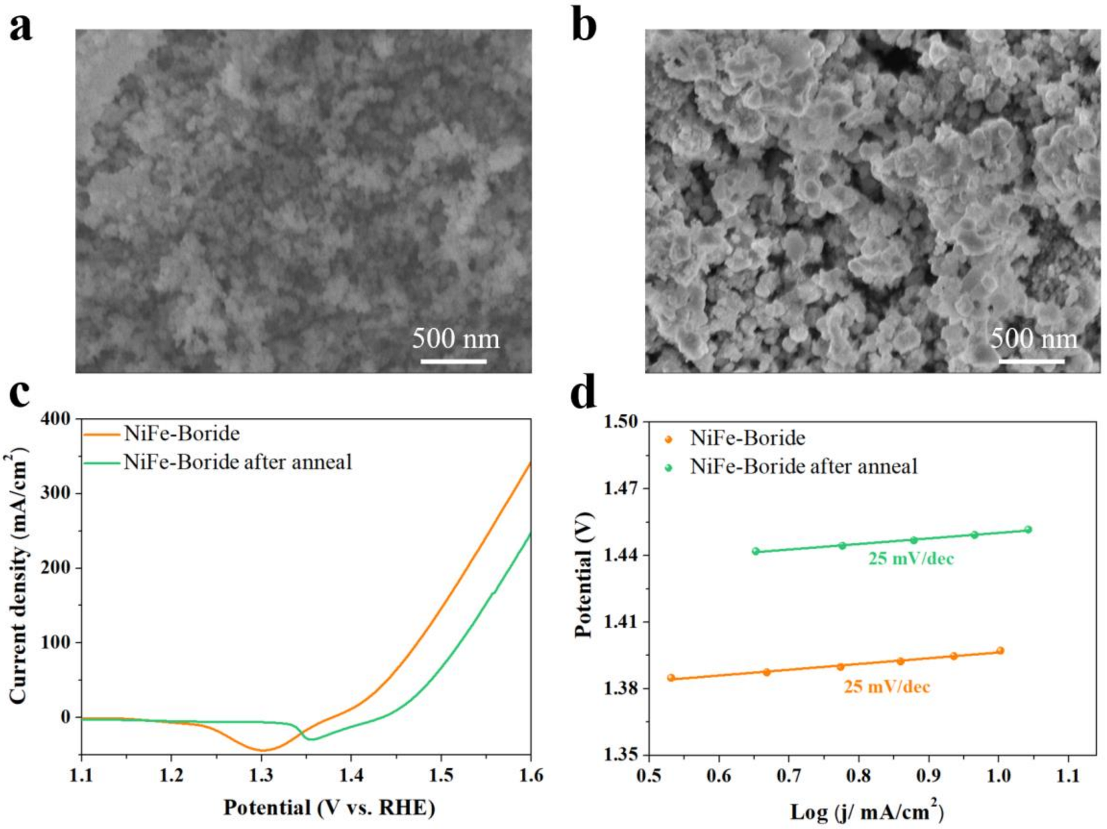

Supplementary Figure 21. Effect of annealing on the morphology and catalytic activity of NiFe-Boride catalyst. SEM images illustrating the surface morphology of (a) fresh and (b) annealed catalyst. (c) OER LSV (*iR*-free) polarization curves for catalysts loaded on Ni foam in a three-electrode configuration in 1 M KOH aqueous electrolyte. (d) The corresponding Tafel plot of catalysts.

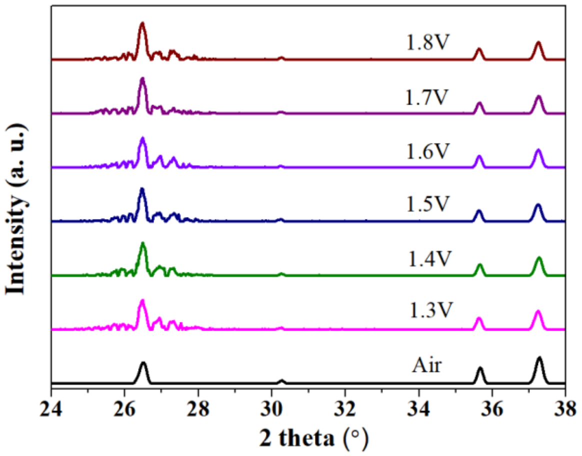

Supplementary Figure 22. *In-situ* SRXRD patterns of the annealed catalyst during the OER test in 1 M KOH.

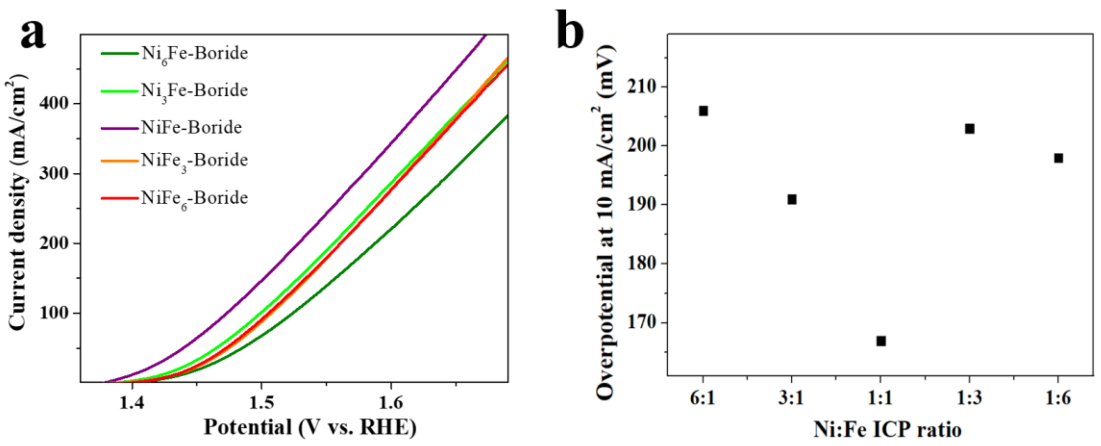

Supplementary Figure 23. Effect of Ni:Fe ICP ratios on the (a) catalytic performance and (b) overpotential of NiFe-Boride catalyst (supported on a Ni foam substrate) at 10 mA/cm^2 in 1 M KOH electrolyte.

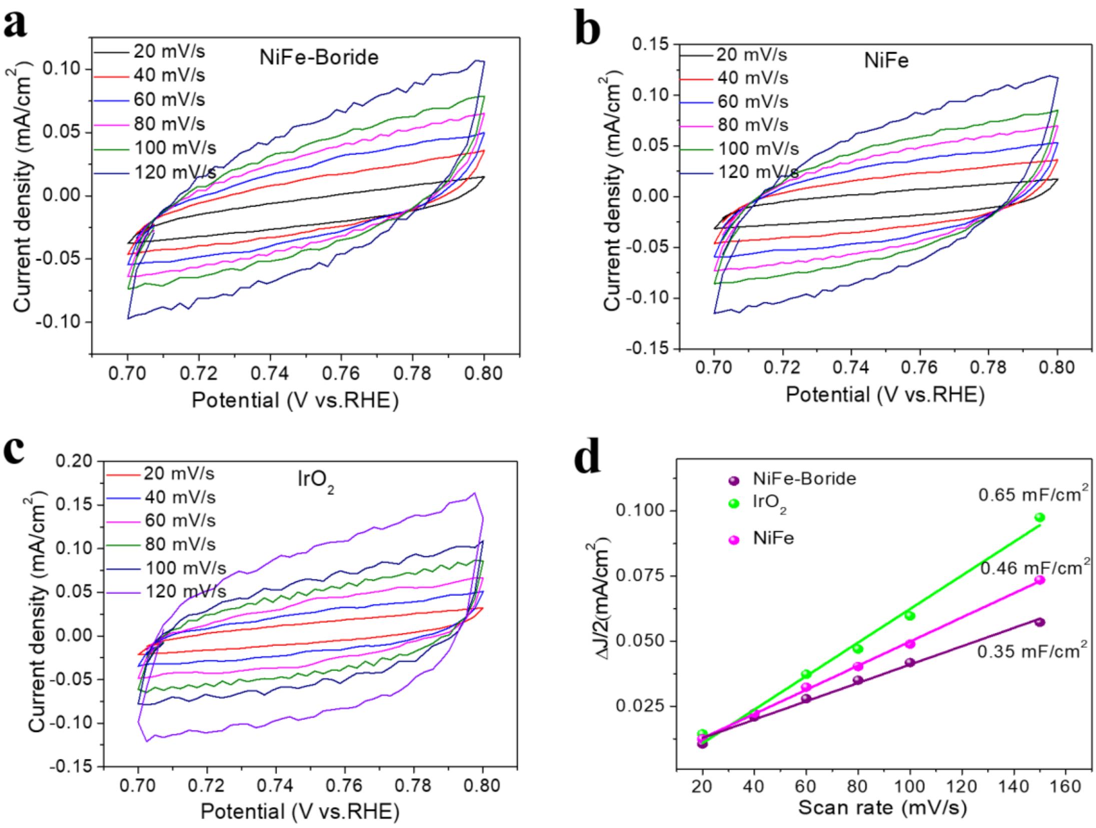

Supplementary Figure 24. Cyclic voltammograms of (a) NiFe-Boride (b) NiFe and (c) IrO_2 catalysts in a voltage range of 0.7 – 0.8 V vs. RHE at scan rates from 20 to 120 mV/s in 1 M KOH aqueous electrolyte. (d) Scan rate dependence of the current densities and their corresponding linear fittings (solid lines).

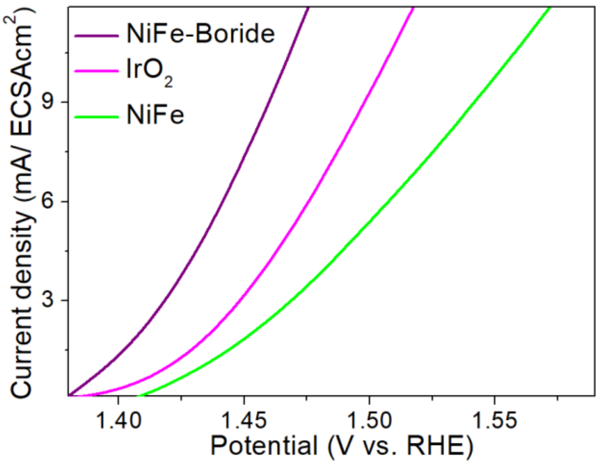

Supplementary Figure 25. LSV normalized by ECSA curves of the catalysts on Ni foam.

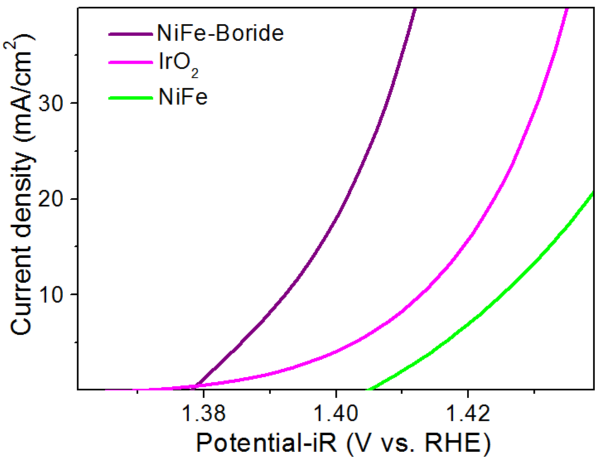

Supplementary Figure 26. The OER polarization curves of NiFe-Boride and control catalysts on Ni foam with identical mass loading after *iR* correction (*R* is the series resistance, obtained from EIS fitting).

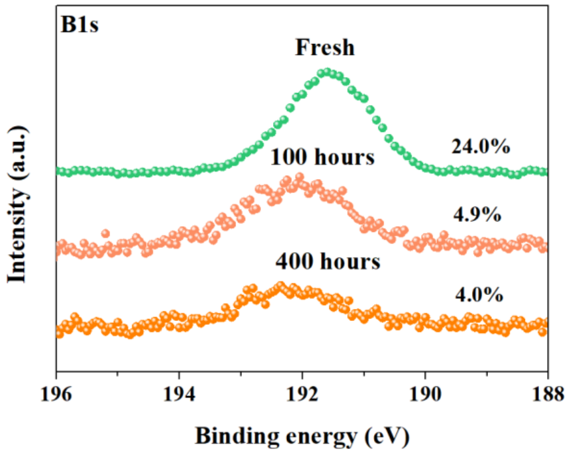

Supplementary Figure 27. High-resolution B 1*s* XPS spectra and atomic ratios of B in NiFe-Boride catalyst before and at various time intervals of the extended OER operation.

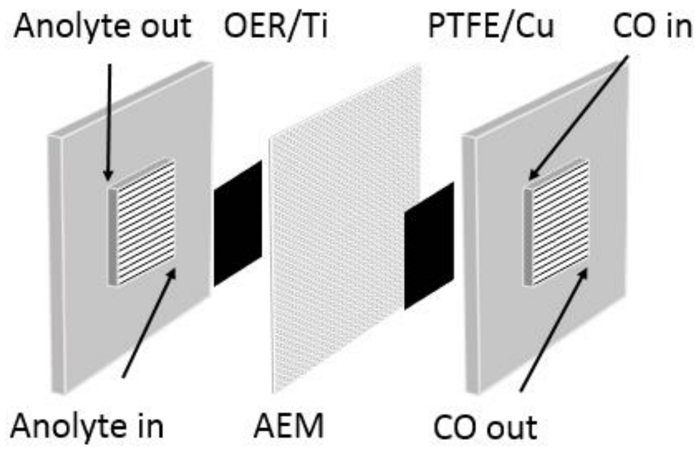

Supplementary Figure 28. A schematic illustration of the MEA system that couple OER at the anode with CORR at the cathode.

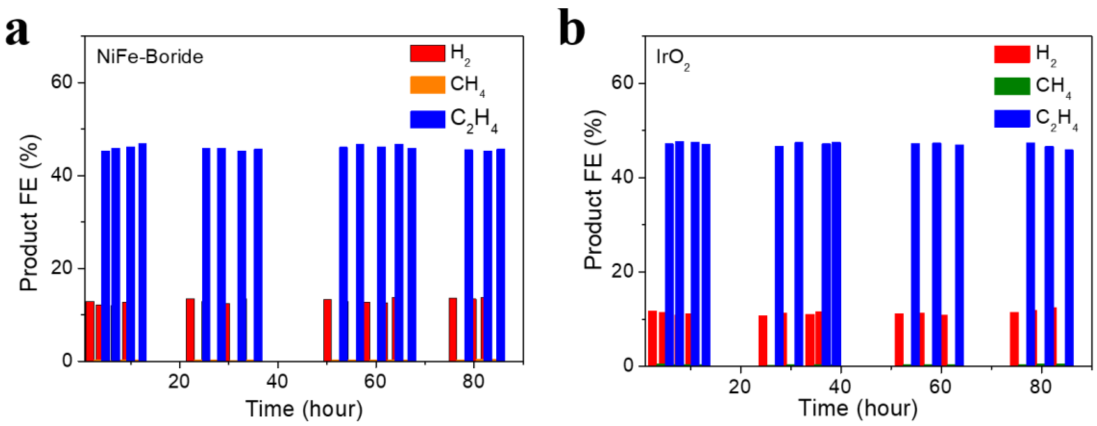

Supplementary Figure 29. Product distribution of CORR for (a) NiFe-Boride and (b) IrO_2 anode catalysts pair with the pure Cu CORR catalyst. The experiments were carried out in a MEA cell using 2 M KOH as electrolyte at the anolyte.

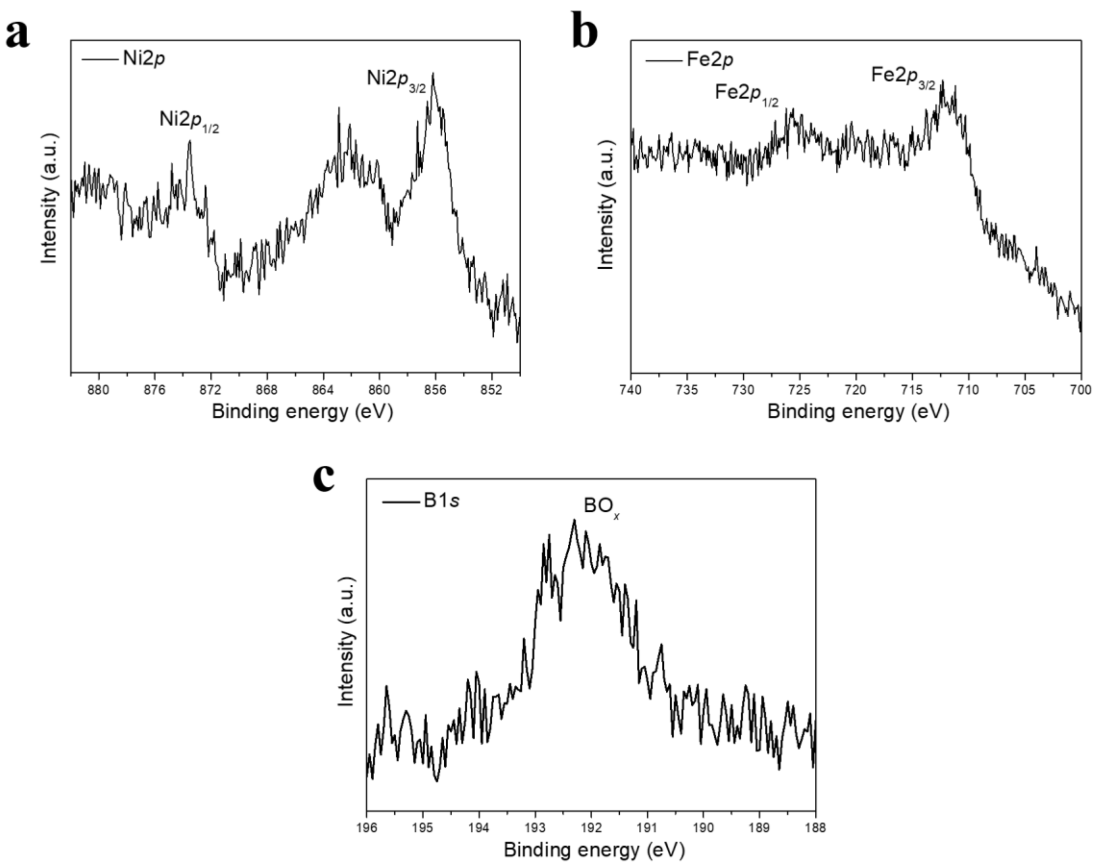

Supplementary Figure 30. The high-resolution (a) Ni 2*p*, (b) Fe 2*p* and (c) B 1*s* XPS spectrum of NiFe-Boride sample after the stability test.

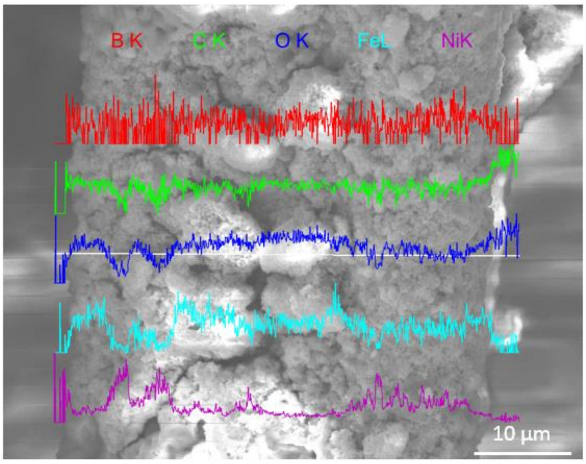

Supplementary Figure 31. The elemental analysis via EDX linear scan for the tested NiFe-Boride catalyst.

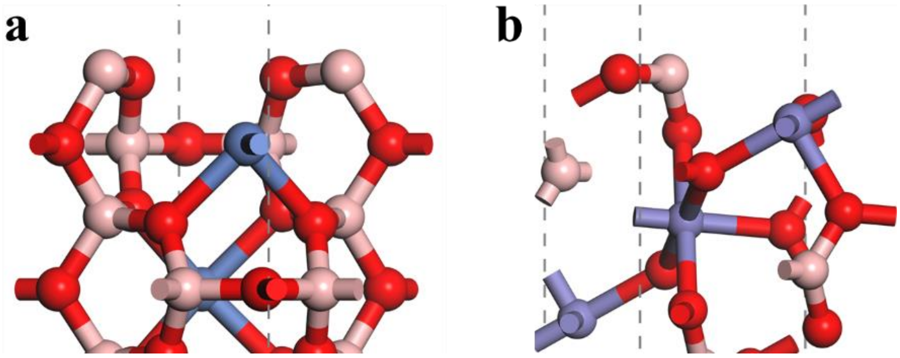

Supplementary Figure 32. Side view of (a) NiB_4O_7 and (b) $FeBO_3$. Pink, red, bice and violet represent B, O, Ni and Fe, respectively.

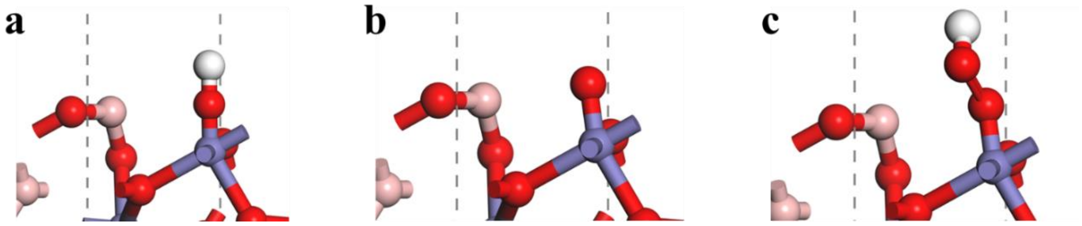

Supplementary Figure 33. Adsorption configurations of (a) *OH, (b) *O, and (c) *OOH on $FeBO_3$ (100) surface. White ball represents H atom.

Supplementary Table 1. The electrochemical-step symmetry index (ESSI) and maximum energy for different OER catalysts.

	ΔG_1 (eV)	ΔG_2 (eV)	ΔG_3 (eV)	ΔG_4 (eV)	ESSI (@1.23V)	G_{max} (@1.23V)
γ-NiOOH	0.02	0.56	0.30	(-0.88)	0.29	0.88
NiB_4O_7	(-0.34)	0.09	0.39	(-0.14)	0.24	0.48
$FeBO_3$	0.33	0.49	0.20	(-1.02)	0.34	1.02

Supplementary Table 2. The lengths of Ni-Ni and Ni-O bonds and coordination numbers extracted from the curve-fitting of Ni *K*-edge EXAFS data.

	Path	CN	R (Å)	σ^2 (Å^2)	ΔE^0 (eV)	R^2
NiFe-Boride	Ni-O	4.6(2)	2.04(4)	0.005(1)	8.6(3)	0.009
Before OER	Ni-Ni(Fe)	3.8(3)	3.09(1)	0.008(5)	3.0(2)	
NiFe-Boride	Ni-O	5.6(1)	2.02(1)	0.005(6)	1.4(2)	0.008
During OER	Ni-Ni(Fe)	8.4(2)	2.98(1)	0.007(3)	-6.3(1)	
NiO	Ni-O	5.5(1)	2.01(1)	0.009(1)	5.4(3)	0.006
	Ni-Ni	10.8(1)	2.91(1)	0.006(5)	-0.4(6)	
NiOOH	Ni-O	5.5(1)	2.01(4)	0.008(1)	-4.1(3)	0.008
	Ni-Ni	5.7(1)	3.09(2)	0.007(2)	-3.1(6)	

Supplementary Table 3. The lengths of Fe-Fe and Fe-O bonds and coordination numbers extracted from the curve-fitting of Fe *K*-edge EXAFS data.

	Path	CN	R (Å)	σ^2 (Å^2)	ΔE^0 (eV)	R^2
NiFe-Boride	Fe-O	4.2(1)	2.23(6)	0.005(1)	-4.1(2)	0.009
Before OER	Fe-Fe(Ni)	2.2(3)	3.22(7)	0.009(2)	-9.9(1)	
NiFe-Boride	Fe-O	5.0(1)	1.92(1)	0.005(5)	-7.8(1)	0.007
During OER	Fe-Fe(Ni)	6.4(1)	3.05(2)	0.007(1)	-6.7(1)	
FeO	Fe-O	5.0(6)	2.11(1)	0.007(2)	2.6(5)	0.004
	Fe-Fe	10.5(1)	3.03(2)	0.009(1)	-6.1(2)	
FeOOH	Fe-O	5.6(1)	1.98(1)	0.007(1)	2.8(1)	0.006
	Fe-Fe	5.7(1)	2.96(2)	0.002(1)	3.0(2)	

Supplementary Table 4. Comparison of the performance metrics for NiFe-Boride and the control catalysts.

Samples	Electrolyte	Overpotential (mV) (at 10 mA/cm^2)	References
**NiFe-Boride**	**1M KOH**	**167**	**This work**
IrO_2	1M KOH	189	This work
NiFe alloy	1M KOH	265	*Nano Lett.* 2020[1]
NiFe LDH/(NiFe)S_x	1M KOH	210	*Electrochimica Acta 2020*[2]
NiFe	1M KOH	189	*Adv. Mater.* 2019[3]
NiFe-LDH@NiCu	1M KOH	218	*Adv. Mater.* 2019[4]
NiFe-LDH	1M KOH	270	*ACS Appl. Energy Mater.* 2019[5]
NiFeO	1M KOH	184 with iR correction	*Energy Environ. Sci.* 2019[6]
NiFeCu	1M KOH	180	*Nat. Commun.* 2018[7]
Au/NiFe	1M KOH	210	*J. Am. Chem. Soc.* 2018[8]
Ni-Fe-O	1M KOH	244	*Adv. Energy. Mater.* 2018[9]
NiFe LDH@NiCoP	1M KOH	220	*Adv. Funct. Mater.* 2018[10]
NiFe LDH	1M KOH	195	*Angew. Chem.* 2018[11]

Supplementary Table 5. The ICP-OES analyses for NiFe-Boride catalysts with various Ni:Fe ratios.

Samples	Fe (ppm)	Ni (ppm)	B (ppm)
Ni_6Fe-Boride	5.76	34.59	12.14
Ni_3Fe- Boride	9.44	28.97	12.33
NiFe- Boride	16.74	17.5	14.53
$NiFe_3$- Boride	29.71	10.66	15.36
$NiFe_6$- Boride	26.54	4.77	16.81

References

1 Cai, W. *et al.* Amorphous versus crystalline in water oxidation catalysis: A case study of NiFe alloy. *Nano Lett.* **20**, 4278-4285 (2020).

2 Zou, Y. *et al.* 3D hierarchical heterostructure assembled by NiFe LDH/(NiFe)S_x on biomass-derived hollow carbon microtubes as bifunctional electrocatalysts for overall water splitting. *Electrochimica Acta* **348**, 136339 (2020).

3 Chen, G. *et al.* An amorphous Nickel-Iron-Based electrocatalyst with unusual local structures for ultrafast oxygen evolution reaction. *Adv. Mater.* **31**, 1900883 (2019).

4 Zhou, Y. *et al.* Exceptional performance of hierarchical Ni-Fe (hydr)oxide@NiCu electrocatalysts for water splitting. *Adv. Mater.* **31**, 1806769 (2019).

5 Teng, X. *et al.* Self-Growing NiFe-Based hybrid nanosheet arrays on Ni nanowires for overall water aplitting. *ACS Appl. Energy Mater.* **2**, 5465-5471 (2019).

6 Qiu, Z., Tai, C.-W., Niklasson, G. A. & Edvinsson, T. Direct observation of active catalyst surface phases and the effect of dynamic self-optimization in NiFe-layered double hydroxides for alkaline water splitting. *Energy Environ. Sci.* **12**, 572-581 (2019).

7 Zhang, P. *et al.* Dendritic core-shell nickel-iron-copper metal/metal oxide electrode for efficient electrocatalytic water oxidation. *Nat. Commun.* **9**, 381, 1-10 (2018).

8 Zhang, J. *et al.* Single-atom Au/NiFe layered double hydroxide electrocatalyst: probing the origin of activity for oxygen evolution reaction. *J. Am. Chem. Soc.* **140**, 3876-3879 (2018).

9 Dong, C., Kou, T., Gao, H., Peng, Z. & Zhang, Z. Eutectic-Derived mesoporous Ni-Fe-O nanowire network catalyzing oxygen evolution and overall water splitting. *Adv.*

Energy Mater. **8**, 1701347 (2018).

10 Zhang, H. *et al.* Bifunctional heterostructure assembly of NiFe LDH nanosheets on NiCoP nanowires for highly efficient and stable overall water splitting. *Adv. Funct. Mater.* **28**, 1706847 (2018).

11 Cai, Z. *et al.* Introducing Fe(2+) into Nickel-Iron layered double hydroxide: local structure modulated water oxidation activity. *Angew. Chem. Int. Ed.* **57**, 9392-9396 (2018).
